# Climate, Environment and Early Human Innovation: Stable Isotope and Faunal Proxy Evidence from Archaeological Sites (98-59ka) in the Southern Cape, South Africa

**DOI:** 10.1371/journal.pone.0157408

**Published:** 2016-07-06

**Authors:** Patrick Roberts, Christopher S. Henshilwood, Karen L. van Niekerk, Petro Keene, Andrew Gledhill, Jerome Reynard, Shaw Badenhorst, Julia Lee-Thorp

**Affiliations:** 1 School of Archaeology, Research Laboratory for Archaeology and the History of Art, the University of Oxford, Dyson Perrins Building, South Parks Road, Oxford, United Kingdom; 2 Evolutionary Studies Institute, University of the Witwatersrand, Johannesburg, South Africa; 3 Department of Archaeology, History, Cultural Studies and Religion, University of Bergen, Bergen, Norway; 4 Division of Geographic, Archaeological and Environmental Sciences, University of Bradford, Bradford, United Kingdom; 5 School of Geography, Archaeology and Environmental Studies, University of the Witwatersrand, Johannesburg, South Africa; 6 Archaeozoology and Large Mammal Section, Ditsong National Museum of Natural History (former Transvaal Museum), Paul Kruger St, Pretoria, South Africa; 7 Department of Anthropology and Archaeology, University of South Africa, UNISA, Durban, South Africa; Universidade do Algarve, PORTUGAL

## Abstract

The Middle Stone Age (MSA) of southern Africa, and in particular its Still Bay and Howiesons Poort lithic traditions, represents a period of dramatic subsistence, cultural, and technological innovation by our species, *Homo sapiens*. Climate change has frequently been postulated as a primary driver of the appearance of these innovative behaviours, with researchers invoking either climate instability as a reason for the development of buffering mechanisms, or environmentally stable refugia as providing a stable setting for experimentation. Testing these alternative models has proved intractable, however, as existing regional palaeoclimatic and palaeoenvironmental records remain spatially, stratigraphically, and chronologically disconnected from the archaeological record. Here we report high-resolution records of environmental shifts based on stable carbon and oxygen isotopes in ostrich eggshell (OES) fragments, faunal remains, and shellfish assemblages excavated from two key MSA archaeological sequences, Blombos Cave and Klipdrift Shelter. We compare these records with archaeological material remains in the same strata. The results from both sites, spanning the periods 98–73 ka and 72–59 ka, respectively, show significant changes in vegetation, aridity, rainfall seasonality, and sea temperature in the vicinity of the sites during periods of human occupation. While these changes clearly influenced human subsistence strategies, we find that the remarkable cultural and technological innovations seen in the sites cannot be linked directly to climate shifts. Our results demonstrate the need for scale-appropriate, on-site testing of behavioural-environmental links, rather than broader, regional comparisons.

## Introduction

The Still Bay (*c*. 77–73 ka) and Howiesons Poort (*c*. 65–59 ka) Middle Stone Age (MSA) lithic traditions of southern Africa are argued to represent major periods of cultural, technological, and subsistence innovation by early *Homo sapiens* [1–3]. Sophisticated heat-treated, pressure-flaked technologies are associated with the Still Bay [4] while the origins of complex hafting technologies and hunting strategies have been associated with the backed stone segments of the Howiesons Poort [5]. Both the Still Bay and Howiesons Poort have also been linked to the earliest examples of material culture associated with symbolically mediated behaviour [6]. However, the factors behind their apparently sudden and widespread emergence and then disappearance remain hotly debated, with demography, sea level, and climate change all argued to have played major roles [7–10].

The punctuated nature of both the Still Bay and Howiesons Poort, and their chronological overlap with the Marine Isotope Stage 5a/4 and 4/3 transitions, respectively, has made climatic variability a particularly attractive focus for researchers. It has been argued that climatic and environmental instability immediately precedes or overlaps these periods, and new behavioural repertoires emerged as buffering mechanisms [11–13]. In contrast, it has also been suggested that climatic and environmental instability are not in phase with human behavioural changes, and cultural innovation instead occurred in hospitable refugia [2]. However, the evidence needed to test these hypotheses rigorously has been lacking. Research in the southern Cape of South Africa, where many of the important MSA sites bearing these industries are found, is currently limited by a general lack of well-understood palaeoenvironmental records with sound chronological control [14]. Where they do exist, they tend to remain spatially and chronologically disconnected from the archaeological sequences they have been used to explain, leading to broad generalisations and untestable correlations.

One means of addressing this problem is the development of high-resolution palaeoenvironmental datasets from *within*, or in close association with, archaeological sequences (e.g. [11]). Although such records can potentially be subject to anthropogenic influence, they are easily dated and can be directly correlated with evidence of early human behaviour. Moreover, archaeological sites offer the potential for the rich and diverse preservation of multiple, independent palaeonvironmental proxies. Stable carbon and oxygen isotope analysis of ostrich eggshell (OES), ubiquitous at MSA and Later Stone Age (LSA) southern African sites, is one such proxy. OES δ^13^C reflects the ambient vegetation consumed by an ostrich during the breeding season [15–18], and δ^18^O, the ostrich’s source water [19–21]. In the southern Cape these parameters are controlled by both seasonality and amount of rainfall as the region is dissected by winter and seasonally-bimodal year-round precipitation zones [22–23]. Plants, which undergo ^18^O enrichment during evapotranspiration [24], are the primary source of an ostriches’ water [25]. Fractionation due to evapotranspiration is negatively correlated to relative humidity [26]. Therefore, OES δ^18^O tracks shifts in relative humidity/aridity [19–21].

**Fig 1:**
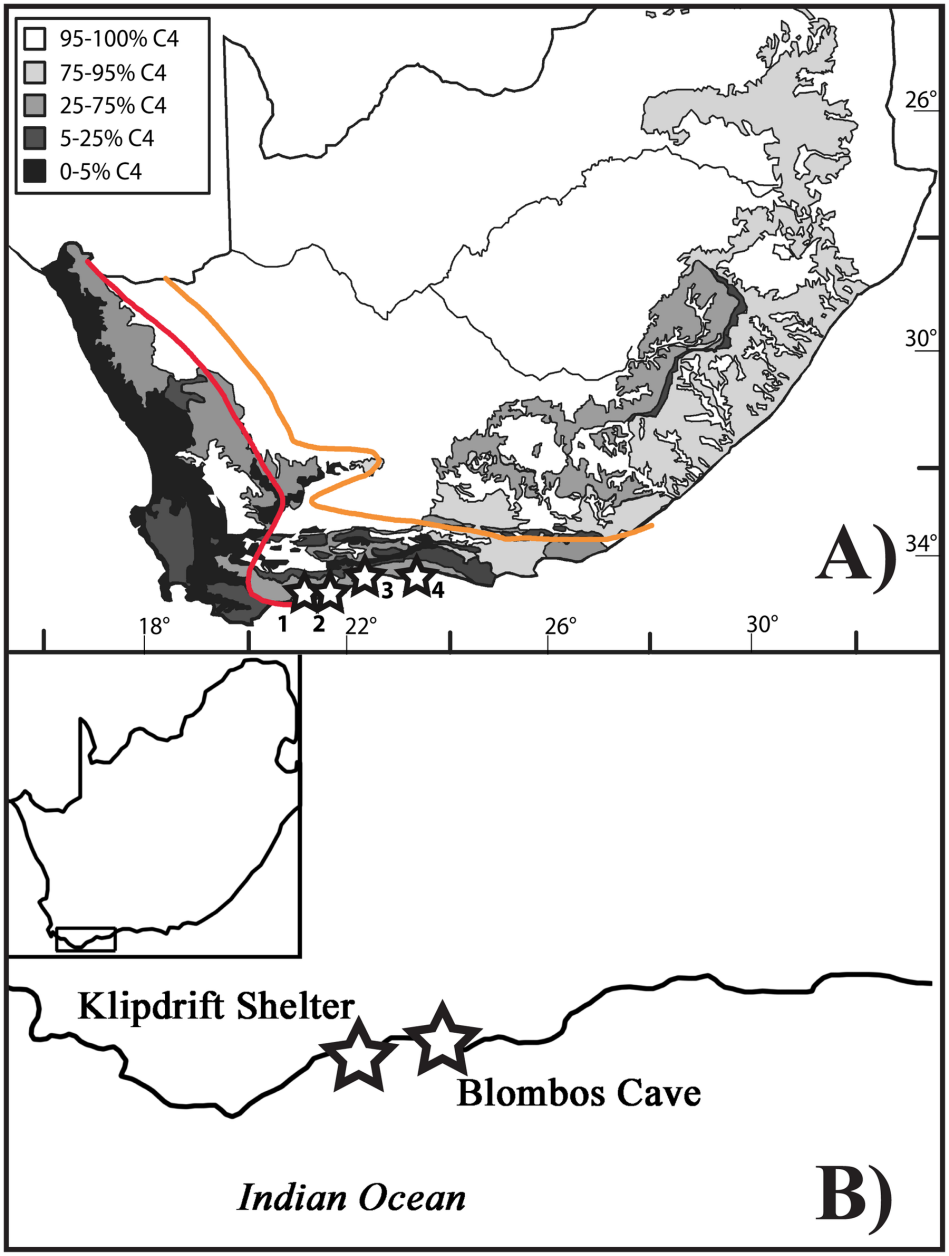
Map of the sites analysed in this study relative to precipitation regime and vegetation distribution. A) The position of sites discussed in this paper (1 = BBC, 2 = KDS, 3 = Nelson’s Bay Cave, 4 = Pinnacle Point) relative to the winter (to the left of the red line), year-round (between the red and orange lines), and summer rainfall (to the right of the orange line) zones of the modern southern Cape coast of South Africa on a map of % C_3_/C_4_ plant species abundances (adapted from Vogel *et al*. [22]). B) A close-up view of the coastline proximate to BBC and KDS.

In this paper, we undertake stable carbon and oxygen isotope analysis of OES, alongside traditional faunal and shellfish environmental proxy analysis, from the MSA occupation levels of Blombos Cave (BBC) (98-73ka) (34°25’S, 21°13’E) and Klipdrift Shelter (KDS) (72–59 ka) (34°27’S, 20°43’E) in order to produce ‘on-site’ records of vegetation, precipitation seasonality, aridity, and sea temperature for the periods of human occupation (Fig 1). A total of 83 and 42 fragments of OES were analysed from BBC and KDS, respectively. In addition to high densities of OES, rich faunal and shellfish assemblages at these sites provide further insight into terrestrial and marine environments of relevance to human ecologies, technologies, and cultural behaviours [27–28]. The archaeological sequences from these sites include both the Still Bay (76.7 ± 4.8–73.3 ± 4.5 ka- [29]) and Howiesons Poort (64.8 ± 4.8 and 59.5 ± 4.6 ka- [28]) traditions, and associated evidence of bone tool technologies, ochre production, and personal ornamentation [6,12,28].

### Stable carbon and oxygen analysis of archaeological OES as a palaeoenvironmental proxy on the southern Cape coast

The ostrich, *Struthio camelus australis*, has been part of Africa’s vertebrate fauna since the Pliocene. Their eggs have been valued by hunter-gatherers of the MSA and LSA, as well as by many ethnographic groups, as indicated by their abundance in southern African archaeological sites, including BBC and KDS. Ostriches are opportunistic mixed-feeders and are one of very few animals known to eat C_3_, C_4_, and CAM vegetation [25,30]. They also show no particular preference for any of these groups and it is instead plant tenderness that dictates ostrich vegetation choice [30]. In order to facilitate adaptation to arid conditions, ostriches are non-obligate drinkers, with limited water excretion, and can survive on green vegetation without drinking for a number of days [25].

δ^13^C values from the inorganic fractions of OES reflect the ambient vegetation consumed by the ostrich in the breeding season in which the eggs were laid [15–17]. The apparent δ^13^C fractionation between plant diet and eggshell CaCO3 (ε*_plant-CaCO3_) is *c*. 15‰ [16–17]. Ostrich preferences for fresh palatable vegetation mean that, while OES δ^13^C will not provide a direct indicator of local plant biomass, it will provide indirect insight into the proportions of C_3_, C_4_ and CAM plants available in the surrounding landscape [18]. The intersection of precipitation-controlled vegetation zones for the last several million years on the southern Cape Coast means that stable isotope analysis of OES can track rainfall dynamics in the past (Fig 1) [19]. Vegetation in the winter rainfall zone comprises mainly C_3_ plants, with relatively low δ^13^C values (globally -24 to -32‰), while vegetation in the year-round rainfall zone includes some C_4_ plants with higher (-10 and -14‰) δ^13^C values [22,31]. CAM plants in the C_3_-dominated winter rainfall zone of the southern Cape coast of South Africa today have ‘C_3_’ δ^13^C, while those in the year-round rainfall region have δ^13^C, spanning C_3_ and C_4_ values [32]. As a result, any increase in CAM taxa will complement the emergence of C_4_ plants in this region.

Oxygen isotope fractionation between body water and CaCO_3_ is about 30‰ (following the standard fractionation from H_2_O to CaCO_3_ [33]). Although OES δ^18^O is influenced by the δ^18^O of rainfall source, given that ostriches obtain most of their body water from plants and recycled metabolic water [25], it is primarily influenced by ^18^O-enrichment in plants due to evapotranspiration [18–21]. Evapotranspiration in leaves leads to preferential loss of ^16^O and ^18^O enrichment in the leaf [24]. The magnitude of this effect is enhanced by low relative humidity [26,34]. As a result, OES δ^18^O strongly reflects the influence of humidity or relative humidity (RH) over and above the regional controls exerted by meteoric water δ^18^O values [19–21]. OES will have more positive δ^18^O under conditions of increased aridity, while lower δ^18^O reflects greater humidity [19–21]. In the context of southern Africa, these evaporation-linked changes will have a much greater effect than any shifts in rainfall source or influence [11,35]. That said, given that the winter rainfall zone is associated with summer drought [19,36], expansion of C_3_ biomes across the southern Cape coast, reflected in lower OES δ^13^C, will likely be associated with increased aridity, and higher δ^18^O, during the ostrich breeding period, which occurs just prior to a region’s rainy season [37].

## Materials and Methods

### Sites

#### Blombos Cave

**Fig 2:**
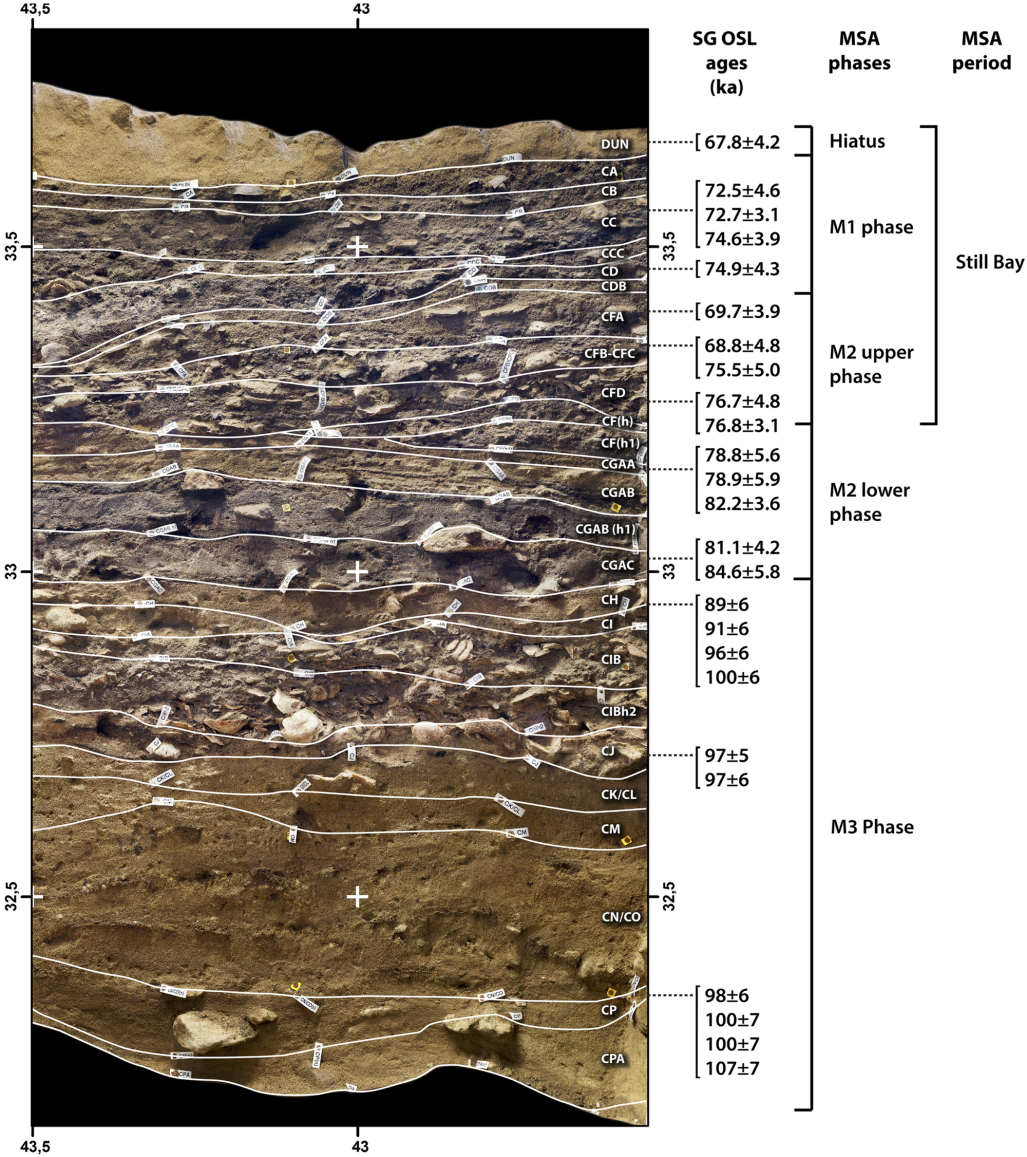
Blombos Cave MSA stratigraphy. MSA Phases M1, M2, and M3, and their associated OSL ages, of BBC (see also [39]).

BBC is located in Blombosfontein Nature Reserve, about 300km east of Cape Town on the southern coast of South Africa (34°25’S, 21°13’E). The cave is positioned on a south-facing cliff *c*. 35m above modern sea level, approximately 100 metres from the present shoreline [38]. BBC is set into the calcified sediments of the Tertiary Wankoe Formation, and the calcareous environment is at least partially responsible for the good preservation of the recovered deposits [38]. In particular, the MSA sequence of BBC represents one of the longest and richest sequences of early subsistence, technological, and cultural innovations by *Homo sapiens* worldwide. The MSA levels of BBC are divided into three phases: M1, M2 (upper and lower), and M3 (Table A in S1 File, Fig 2).

The M1 and upper M2 phases contain Still Bay-type bifacial foliate points in association with evidence for shell beads, formal bone tools, engraved bone and ochre. Thermoluminescence (TL) dates from burnt lithics, Electron-Spin Resonance (ESR) age estimates on faunal tooth enamel [40], and four Multi-Grain Optically Stimulated Luminescence (OSL) ages from the M2 phase previously indicated a range between 80 and 60 ka (39) (Table A in S1 File). More recent Single Grain Optically Stimulated Luminescence (SG-OSL) assessments, however, now suggest that these phases began no earlier than 75.5ka and ended no later than 67.8 ka [29] (Table B in S1 File) (Fig 2).

The Still Bay-type bifacial points from the M1 and upper M2 phases of BBC are made from silcrete, quartzite, and quartz. A macrofracture study of these points shows that while some were used as spear points, others probably served as multifunctional tools [41]. Approximately half of the silcrete points were heat treated and finished using pressure-flaking methods [42]. More than thirty formal bone tools have also been recovered from the Still Bay levels [38,43]. Some of the bone tools were polished after being shaped and scraped and have been suggested to be hafted projectile points [44].

More than 2,000 pieces of ochre have been recovered from the Still Bay phases. Two ochre pieces with deliberately engraved cross-hatched patterns come from the M1 phase [45]. The designs clearly result from deliberate action and have been considered among the earliest abstract representations [45–46]. A further six engraved ochre pieces from these phases have been recovered [46]. Microscopic analysis of a bone fragment has revealed that it was also deliberately marked with eight parallel lines [47]. The discovery of 65 *Nassarius kraussianus* shell beads in the Still Bay phases of BBC is synonymous with personal ornamentation, and use-wear analysis indicates they were hung on a cord or sinew [44,48].

Although Multi-grain OSL and a Thermoluminescence date on burnt silcrete exist for the lower M2 phase [49] (Table A in S1 File), here we use a more recent SG-OSL determination of 77 ± 3 ka to date this phase (Table B in S1 File). Bone technology, bifacial points, and shell beads are absent from the lower M2 phase. The intensity of cultural deposits is low in this phase, without any evidence for the deliberate engraving of ochre pieces, and human occupation of the site was likely of short duration and representative of small group sizes at this time [38,50].

The similarity of SG-OSL ages between the different layers of phase M3 suggests that the deposits accumulated over a short time interval in the middle part of Marine Isotope Stage (MIS) 5 between about 107 and 90 ka (MIS 5c to 5b) (Table A and Table B in S1 File). There is no support for a significant difference in timing between layers CJ and CH/CI as previously suggested by Jacobs *et al*. [51], following re-analysis of sample ZB5 [29]. An SG-OSL date from the CQ hiatus sand layer of 143.2 ± 4.5 ka provides a *terminus post quem* for initial occupation of the site. Lithics are abundant in phase M3 though no bone tools have yet been recovered from this phase [50]. Modified ochre is common, with eight slabs demonstrating deliberate engravings [46]. Finds of two *Haliotis midae* shells containing a pigment-rich compound and associated artefacts forming part of a toolkit has led to claims that the site was used as an ochre processing workshop during part of this phase [39].

OES has been excavated from all three phases of BBC but no engraved specimens have been found.

#### Klipdrift Shelter

The Klipdrift Cave complex is a wave cut platform located 19 metres above sea level in a steep quartzite cliff (34°27.0963’S, 20°43.4582’E) in the De Hoop Nature Reserve, 12–15 metres from the Indian Ocean and 45 kilometres west of BBC. KDS is a *c*. 7 metre deep shelter, separated from a larger, western cave area by a promontory. KDS was first excavated in 2011 with subsequent seasons in 2012 and 2013.

**Fig 3:**
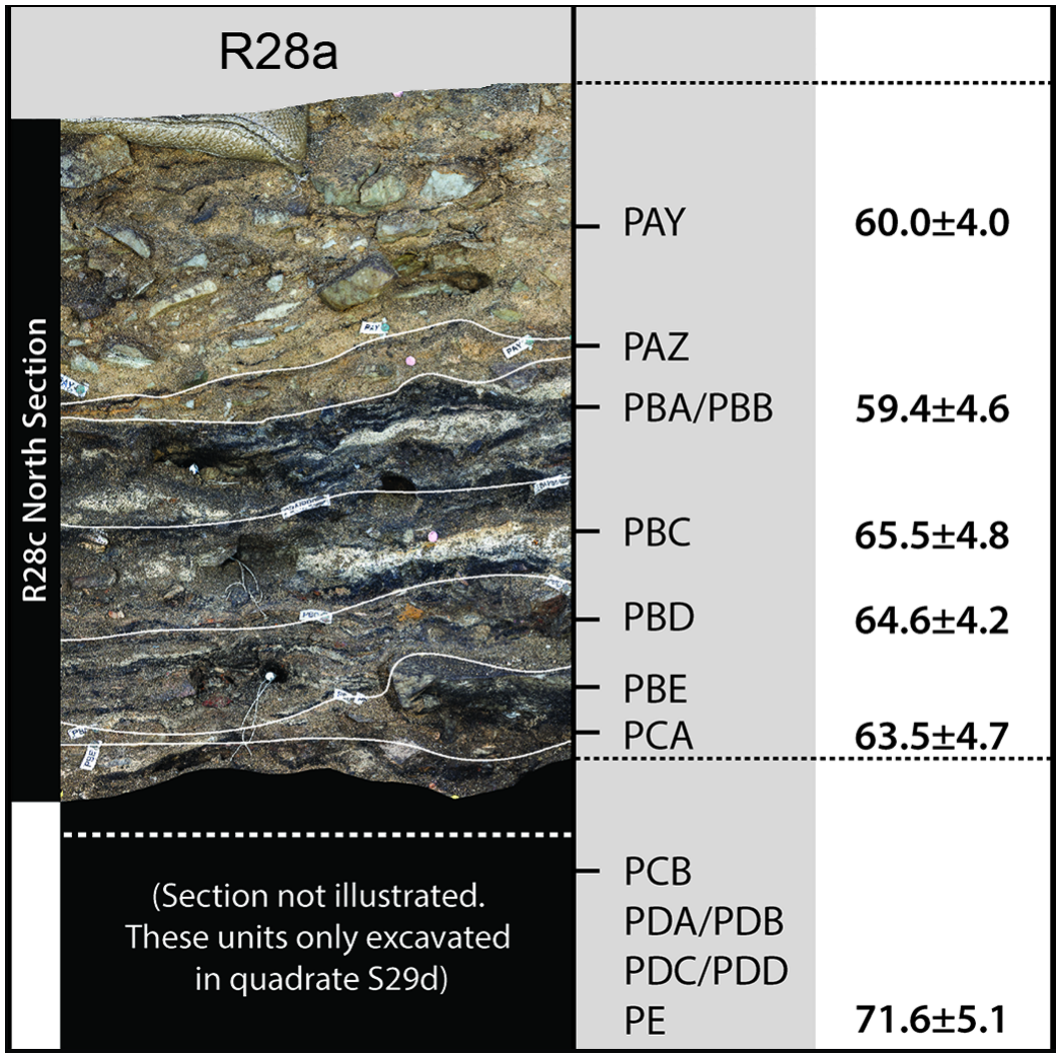
Klipdrift Shelter MSA stratigraphy. MSA layers, and associated OSL dates, for KDS (see also [28]).

The uppermost dated layer yields an SG-OSL age of 51.7 ± 3.3 ka, the middle layers containing Howiesons Poort-type industries range from 65.5 ± 4.8 to 59.4 ± 4.6 ka and the lowermost excavated, anthropogenically sterile layers give an age of 71.6 ± 5.1 ka [28]. Although layer PDA is dated to between *c*. 71.6 and 63.5 ka, the remainder of the OES sequence is dated to between *c*. 65.5 and 59.4 ka (Fig 3) (Table B in S1 File).

While the KDS tools are typical of the Howiesons Poort of southern Africa, three main technological phases can be observed within the sequence [28]. The lowermost phase (PCA, PBE) is characterised by the predominant exploitation of silcrete for blade production, the prevalence of notched tools, the presence of strangulated blades and of highly standardized truncated blades. The following phase (PBC, PBA/PBB) is marked by an increase in quartz exploitation that becomes the most common raw material, while backed tools, including typical segments constitute the main tool group. The uppermost phase (PAY) is defined by the predominance of quartzite, an increase in blade size, the emergence of an independent and structured flake production based on a Levallois concept, a decrease in backed tools, and the presence of a few unifacial points. This phase could be interpreted as a transitional layer towards the post-HP [28].

356 pieces of ochreous material have been recovered from KDS. By mass, PBE has the highest concentration of red ochre in the assemblage (847.6g), although much of this consists of finely processed pieces weighing less than 0.1g. PBE also represents the highest concentration of red ochre, derived from a more limited focus on certain geological types, and is argued to represent the deliberate processing of large amounts of ochre for very specific purposes. By contrast, PBC exhibits the widest geological variability in the sequence [28]. Ninety-five fragments of deliberately engraved OES have also been recovered from layers PAY to PCA (3.8% of the total number of OES fragments), with the majority coming from PBC and PBD [28].

### Stable carbon and oxygen isotope analysis of OES

OES fragments were excavated and bagged from stratigraphic layers at BBC and KDS where present. Sampling of the resulting OES collections was focused on stratigraphic layers which had well-recorded archaeological data for subsistence, technology, or material culture.

OES isotopic values represent a very short period of ostrich plant consumption, and values can therefore vary between eggs laid at marginally differed times of year [15–17,52]. Furthermore, a number of female ostriches can contribute to a nest in any one year [53]. Each female may have slightly different dietary habits and, therefore, δ^13^C and δ^18^O values. To take into account this variation, the maximum number of available OES fragments were measured from each stratigraphic layer sampled (at least 7 and up to 12). This is a great advance on previous studies [54] and follows Ecker *et al*. [20] and Lee-Thorp and Ecker [21]. The samples analysed in this study are listed in Table C and Table D in S1 File. δ^13^C and δ^18^O values from each site were also examined in bivariate plots to avoid including two samples from the same egg in statistical analysis (Figure A and Figure B in S1 File).

Concerns have been raised regarding the movement of small OES fragments through an archaeological sequence as a result of burrowing action or bioturbation [55]. However, where finely excavated sequences have been available for the MSA, protein diagenesis dates from OES have been shown to complement those from other methodologies [55]. The refined, modern excavation methodologies applied during the new excavations at BBC and KDS ensure that the OES sampled in this study are from well-understood, firm contextual settings with no evidence for stratigraphic disturbance [28,56]. While Johnson *et al*. [16–17] demonstrate only small shifts in δ^13^C values of the inorganic and organic fractions with heating, obviously burnt samples are avoided where possible in this study.

The curation and long-distance exchange of ostrich eggshell, notably in the form of beads, has been demonstrated ethnographically [57–58]. However, no evidence for this so far exists in the MSA or LSA records of southern Africa. Nevertheless, only plain fragments, and no beads, with no evidence for engraving or decoration were used in this study in order to avoid potentially curated and transported artefacts (with none existing at BBC regardless). In addition, given that the southern Cape coast provides an excellent environment for ostriches, and OES frequency is correlated with frequencies of grazing animal taxa at KDS [28,59], it seems likely that the OES sampled is representative of local subsistence opportunities and ostrich availability in the past. The OES analysed in this study is therefore likely representative of local, or at least, regional conditions.

Samples were cleaned on all edges using an air abrasion system. 5 x 5 millimetre pieces of OES were then removed from each sample using a craft knife. The ‘interior’ edge of these fragments was then sampled using a diamond-tipped drill. Samples were weighed out to approximately 0.150 mg using a Sartorius CP2 P microbalance, with the resulting powder transferred into glass vials with sealed lids. These vials were then placed in a heated tray maintained at 70°C. Following reaction with 100% Phosphoric Acid, gases evolved from the samples were analysed to stable carbon and oxygen isotopic composition using a Thermo Gas Bench 2 connected to a Thermo Delta V Advantage Mass Spectrometer in the Stable Light Isotope Facility, University of Bradford. Carbon and oxygen isotope values were compared against international standards registered by the International Atomic Energy Agency. Replicate analysis of an internal OES standard suggests that analytical error is *c*. ± 0.1‰ for δ^13^C and ± 0.2‰ for δ^18^O.

Statistical regression analyses were undertaken to discern the statistical correlation between δ^13^C and δ^18^O at both sites. The significance of δ^13^C and δ^18^O variation by layer and site was determined by ANOVA comparative tests for each isotope. Where variance was found to be significant, this was combined with a post-hoc Tukey-corrected pair-wise comparison to determine which layers were significantly different from each other. Given that ANOVA tests, and post-hoc Tukey comparisons, work best when even samples sizes are maintained, when applying this measure by site, the BBC dataset was split in two. Data from levels CC, CD, CF and CI were treated as BBC 1 and levels CJ, CK, CL and CN/CO were treated as BBC 2. All statistical analyses were conducted using the free programme *R* software.

### Faunal analysis

Mammalian fauna from excavations at BBC spanning 2001–2010 from layers CH to CL (Phase M3) were analysed by SB. JR analysed further specimens from the 2011 and 2013 excavation seasons at BBC from layers CF to CA (the M1 and Upper M2 phases). Layer CG (the Lower M2 phase) was not analysed. A total of 3,783 specimens from the M3 and 948 specimens from the M1 and Upper M2 phases were identified to at least the class level. We also examined fauna from the 2011 and 2012 excavation seasons at KDS from layers PDC to PAU. Of the 35,864 specimens recovered from KDS, 2,266 (6.3%) could be identified to at least the class level. Piece-plotted specimens and faunal remains recovered from coarse fraction screened through 3mm sieves were analysed at both sites. Sample sizes for both BBC and KDS were relatively small because of the fragmented nature of the assemblages. For example, at BBC only 2.9% of the 32,546 specimens recovered from the M1 and Upper M2 were identifiable.

The assemblages were analysed following Driver [60] and Klein and Cruz-Uribe [61] using the comparative faunal collections of the Ditsong National Museum of Natural History (formerly the Transvaal Museum) in Pretoria. Only mammals the size of, or larger than, the Cape dune molerat (*Bathyergus suillus*) are included in this analysis. Taxa denoted as ‘cf.’ are included in this study. We use Skinner and Chimimba [62] to categorise ungulates into grazers, browsers and mixed-feeders. In our analysis, eland (*Tragelaphus oryx*) are classified as mixed-feeders due to their tendency to sometimes consume grass during summer [63]. Extinct taxa such as the blue antelope (*Hippotragus leucophaeus*), giant buffalo (*Syncerus antiquus*) and Cape horse (*Equus capensis*) were assigned dietary categories based on previous research [64–65]. Due to evidence of trophic flexibility of eland [63,65] and *Raphicerus* [66]–two of the most prominent bovids at BBC and KDS–we combine browsers and mixed-feeders.

### Shellfish analysis

The BBC shellfish data consist of an enlarged sample (from an additional 7 quadrats) to that published previously [38]. The BBC shellfish data are from 261.4kg of shell fragments– 76.6kg from layers CF to CA (M1 and Upper M2 phases), 17.8kg from the CG layers (Lower M2 phase) and 167.1kg from layers CP to CH (M3 phase). These comprise a minimum number of 16,861 specimens (MNI). The enlarged sample did not significantly change densities reported previously. The KDS shellfish data used here are from Henshilwood *et al*. [28] and are from a 29kg sample with a total MNI of 999. Shellfish were analysed according to the methods outlined by Henshilwood *et al*. [28, 38].

## Results and Discussion

### OES δ^13^C and δ^18^O sequence and chronology

**Fig 4:**
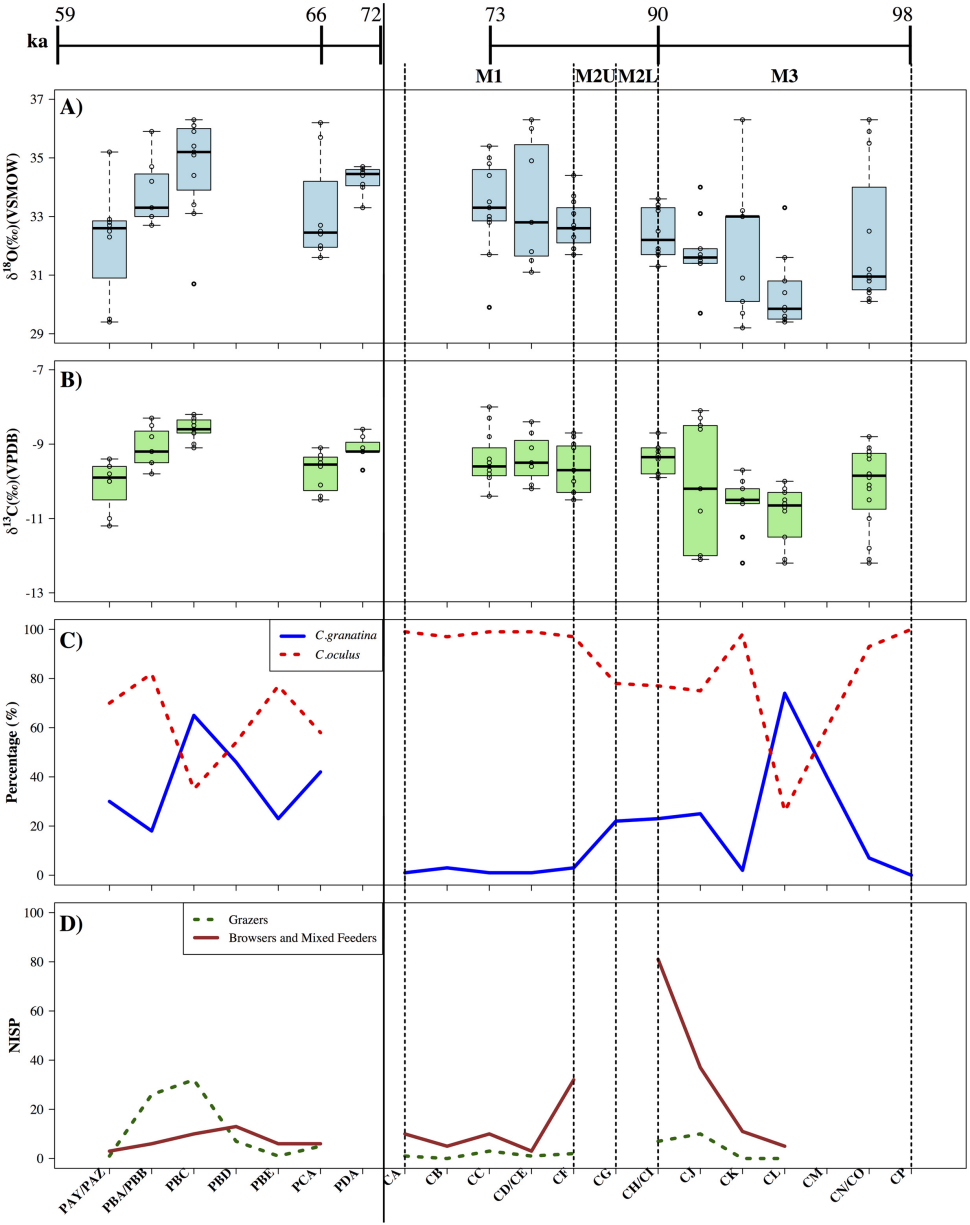
Palaeoenvironmental proxy evidence from Blombos Cave and Klipdrift Shelter. A) δ^18^O and B) δ^13^C measurements from ostrich eggshell (OES), C) relative proportion of grazing versus browsing/mixed feeding taxa in the mammalian assemblage, D) relative proportions of cold water inhabiting *Cymbula granatina* and relatively warmer water indicating *C*. *oculus*. Approximate chronometric ages for the sequence, based on Single Grain Optically-Stimulated Luminescence estimates available from BBC and KDS, are also shown (28–29,39,51)(Table B in S1 File). Bold vertical lines indicate the division between the site sequences. Dashed vertical lines separate the labelled phases of BBC. OES from BBC layers CD and CI has been grouped under CD/CE and CI/CH, respectively, to facilitate comparison with faunal and shellfish material.

OES δ^13^C and δ^18^O data from BBC and KDS are shown in Fig 4 (Table E and Table F in S1 File). An ANOVA statistical test, including post-hoc Tukey HSD corrected pairwise comparison, of the δ^13^C data from BBC indicates that δ^13^C from layers CJ, CK, CL and CN/CO forms a different population to that from layers CC, CD, CF and CI and also the KDS layers (F(2,122) = 20.75, p<0.05)(Table G in S1 File) (Fig 4). ANOVA and Tukey pairwise comparison tests by layer within the whole BBC dataset confirm this trend, with CL and CC, CL and CD, and CL and CI proving to be significantly different from each other (F(7,75) = 3.87, p<0.05)(Table I in S1 File). An ANOVA test of δ^18^O variance at BBC shows that, like δ^13^C, δ^18^O values from layers CJ, CK, CL and CN/CO are significantly different to δ^18^O values from layers CC, CD, CF and CI and the KDS group (F(2,122) = 15.76, p<0.05)(Table H in S1 File). ANOVA and Post-hoc Tukey pairwise comparison tests by layer across these two groups support this trend, indicating layers CL and CC, CL and CD, and CL and CF to be significantly different from each other in terms of δ^18^O (F(7,75) = 3.22, p<0.05)(Table J in S1 File). Regression analysis suggests a weak correlation between δ^13^C and δ^18^O at BBC (Multiple R-squared = 0.25, p<0.01, adjusted R-squared = 0.24, p<0.01).

ANOVA testing demonstrates significant δ^13^C differences between layers (F(4,37) = 13.63, p<0.05) at KDS (Fig 4). Post-hoc Tukey multiple comparisons drew out differences between layers PBA/PBB and PAZ, PBC and PAZ, PDA and PAZ, and PCA and PBC as statistically significant (p<0.05)(Table K in S1 File). This corresponds in Fig 2 to a decrease in δ^13^C from layer PDA to PCA before a significant increase and peak at PBC before a decline in layer PAZ. At KDS, ANOVA testing indicates greater δ^18^O difference between layers than within them (F = (4,37) = 3.78, p<0.05) with post-hoc Tukey comparisons suggesting that layers PBC and PAZ are significantly different from each other (p<0.05)(Table L in S1 File). Inter-layer statistical differences are fewer than for δ^13^C, though regression analysis, and visual comparison in Fig 4, suggests stronger correlation between δ^13^C and δ^18^O at KDS than at BBC (Multiple R-squared = 0.51, p<0.01, Adjusted R-squared = 0.49, p<0.01).

The chronology of the OES sequence presented here is based on 23 and 6 existing Single-Grain Optically Stimulated Luminescence (SG-OSL) dates from BBC and KDS, respectively [2,28,39,51] (Fig 4) (Figs 1 and 2) (Table B in S1 File). Taken together, these dates indicate that the BBC OES sequence covers the period 98 ka (date from the CP Upper layer) to 73 ka (layer CC). Significant δ^13^C and δ^18^O enrichment, between layers CJ to CI, had certainly occurred by the end of Phase M3 *c*. 90 ka [29,39,51]. At KDS, SG-OSL dates indicate that substantial fluctuations seen in δ^13^C and δ^18^O between layers all occur within the timespan of *c*. 72 to 59 ka [28].

### Changes in vegetation, precipitation source, and precipitation amount on the southern Cape coast of South Africa (98-59ka)

Late Pleistocene δ^13^C records from faunal tooth enamel and speleothems on the southern Cape coast of South Africa have been used to track changes in the regional proportion of C_3_ and C_4_ plant taxa and, indirectly, shifts in the seasonality of rainfall [11,67]. While further East, at Nelson’s Bay Cave, Sealy showed little change in the local proportions of these taxa over the last 20,000 years [67], closer to BBC and KDS, at Pinnacle Point, Bar-Matthews *et al*. [11] argued that the considerable shifts in speleothem δ^13^C were indicative of substantial changes in seasonal rainfall influence between 90 and 53 ka.

OES δ^13^C data from the sites of BBC and KDS show significant change over the period 98–59 ka but, compared to changes in δ^13^C enrichment seen in the nearby Pinnacle Point speleothem record, they are muted. This difference may be due to the fact that ostrich diets will not necessarily fully reflect shifts in C_4_ plant availability at this time [21]. In addition, higher OES δ^13^C can record aridity-dependent changes in C_3_ grass δ^13^C, or CAM presence, associated with changes in local aridity, rather than rainfall seasonality [19,21].

As with the OES δ^18^O measured here, δ^18^O values from the Pinnacle Point speleothem record also show fluctuations during this time that have been interpreted as being driven by changes in rainfall source [11]. Compared to the speleothem record, however, our OES data demonstrates more dramatic δ^18^O fluctuations between 98–59 ka that cannot be accounted for by winter and summer rainfall ‘source’ effects even at their extremes (following West *et al*. [35]). This is unsurprising given that while speleothem δ^18^O is reflective of changes in groundwater, and therefore predominantly precipitation, OES δ^18^O is strongly influenced by the evaporative potential of plant transpiration in the region [17–21].

Lowest OES δ^13^C and δ^18^O measurements occur at the beginning of the BBC sequence, suggestive of humid, winter rainfall conditions at this time. Between at least 90 ka to *c*. 73 ka the sequence demonstrates higher δ^13^C and δ^18^O, indicative of increasing aridity and, potentially also, increased year-round rainfall influence or aridity-linked CAM presence. From *c*. 72 to 59 ka at KDS substantial fluctuations in δ^13^C and δ^18^O values imply that this was a period of great instability in plant evapotranspiration, humidity/aridity, and seasonal rainfall dynamics.

### Comparison of OES δ^13^C and δ^18^O, faunal, and shellfish records

Bar-Matthews *et al*. [11] argue that the coincidence of change in speleothem δ^13^C and δ^18^O between 97 and 68 ka, and a substantial period of climatic instability between 68 and 60 ka, at Pinnacle Point, is illustrative of increasing aridity during times of greater summer rainfall influence on the southern Cape coast. This contrasts with suggestions that the winter rainfall zone expanded across the southern Cape coast during glacial periods [23]. Our OES record also demonstrates simultaneous changes in δ^13^C and δ^18^O between *c*. 100 and 59 ka. The combination of our data with faunal and shellfish environmental proxy datasets, however, enables us to tease apart the primary influences on OES δ^13^C on the southern Cape coast through time.

At BBC, enrichment in δ^13^C follows increased proportions of the warm water shellfish indicator species *Cymbula oculus* (Fig 4). Warmer waters on the southern Cape suggest suppressed upwelling, lower influence of easterlies and concomitant increasing influence of westerly winds and winter rainfall [68]. Just prior to OES δ^13^C enrichment at the end of Phase M3 there is also an increase in browser/mixed feeder, rather than grazer, taxa perhaps associated with C_3_ presence and winter rainfall (Fig 4) (Table M and Table N in S1 File). Increased OES δ^18^O indicates increased aridity at this time and, overall, it is likely that OES δ^13^C enrichment also documents this increased aridity, perhaps in the form of increased CAM presence, and increased summer drought strength in the winter rainfall zone, rather than an increase in C_4_ vegetation and summer rainfall influence at this time.

By contrast, enrichment in OES δ^18^O, indicative of increased aridity, at KDS during MIS 4 is associated with increased relative proportions of the cold-water shellfish taxa, *Cymbula granatina* (Fig 4). Colder waters suggest an increased prevalence of near-shore upwelling, easterly winds and summer rainfall at this time which could lead to increased C_4_ presence in the region [68]. Furthermore, unlike BBC, enrichment in OES δ^13^C in layer PBC is accompanied by higher numbers of grazing taxa that dominate the faunal assemblage at this time (Fig 4) (Table O in S1 File). This indicates that in this instance OES δ^13^C enrichment is indicative of increased C_4_ grassland resources in the region. As a result, it seems that the relationship between changing aridity and rainfall regime influence is more complex and context-specific than has previously been suggested [23].

### Testing links between environmental and archaeological change at Blombos Cave and Klipdrift Shelter

**Fig 5:**
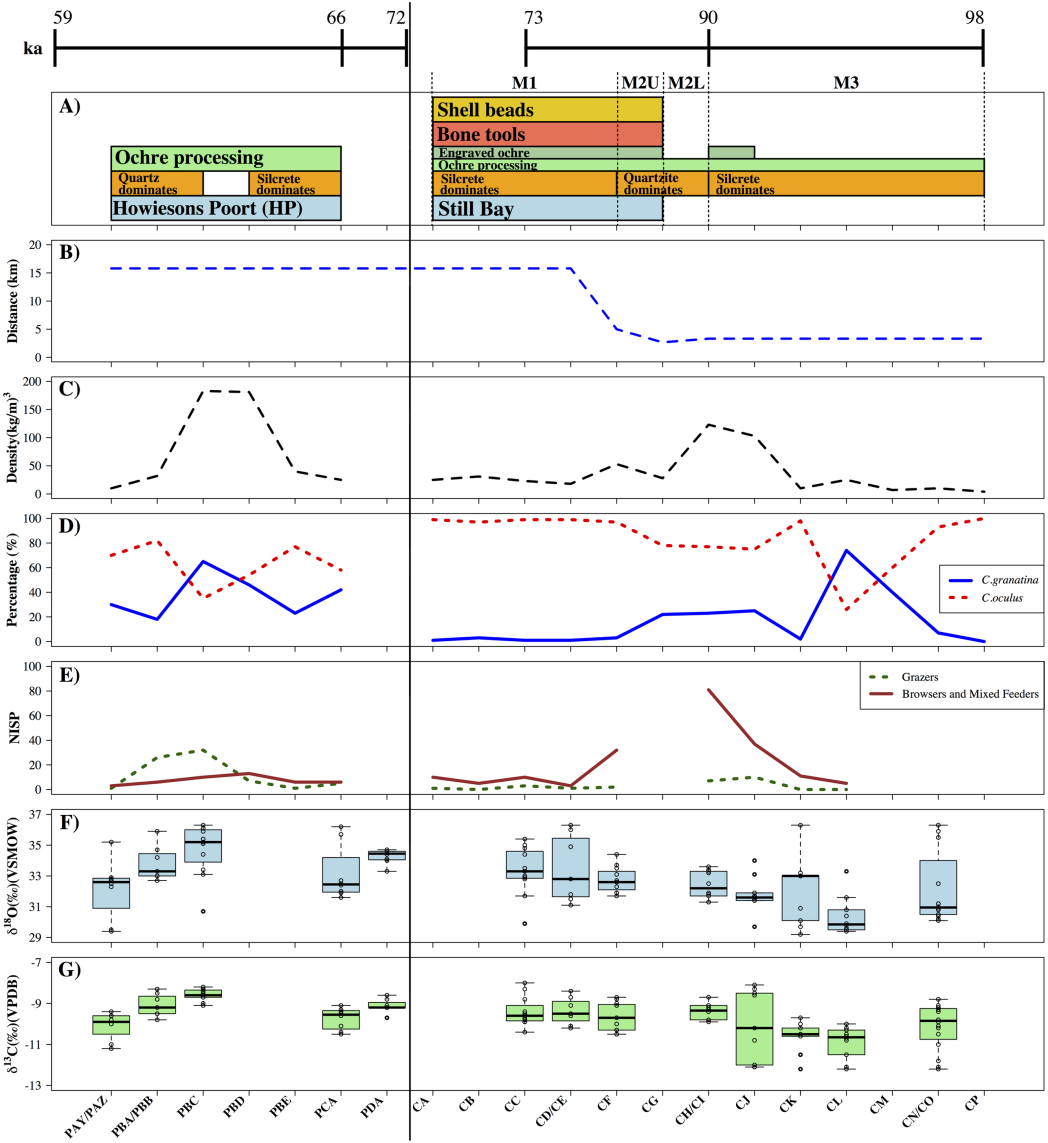
Archaeological sequences and palaeoenvironmental proxies from Blombos Cave and Klipdrift Shelter. Comparison of A) technological and cultural records from BBC and KDS with B) estimated distance of the sites from the coast [Fisher et al., 2010], C) shellfish density, D) relative proportions of cold water inhabiting *Cymbula granatina* and warmer water indicating *C*. *oculus*, E) relative proportion of grazing and browsing/mixed-feeding mammalian taxa (Table M, Table N, Table O in S1 File), F) OES δ^18^O and G) OES δ^13^C. Approximate chronometric ages for the sequence, based on Single Grain Optically-Stimulate Luminescence estimates available from BBC and KDS, are also shown [28, 29,39,51](Table B in S1 File). OES from BBC layer CI has been grouped under CI/CH to facilitate comparison with faunal and shellfish material.

Comparison of OES δ^13^C and δ^18^O, faunal proxy, and shellfish datasets also provides some information regarding human subsistence responses to environmental change at BBC and KDS. The shift to higher OES δ^13^C and δ^18^O at BBC by the end of Phase M3 (*c*. 90 ka), and increased winter rainfall influence, indicated by changing proportions of browsing taxa and *Cymbula oculus*, is associated with increased shellfish densities, increased numbers of large mammal taxa in the faunal assemblage, and increased subsistence diversity, including increased tortoise, small mammal, fish and marine mammal exploitation [27,69–71] (Fig 5). Consistent with the OES δ^13^C and δ^18^O record, increasingly dry conditions are suggested from the faunal assemblage and may indicate humans adjusted their subsistence breadth in the face of climate change. From *c*. 90–78 ka and from 77 ka onwards the coastline has been modelled as being *c*. 8 km and 4km away from the site, respectively [9]. The increased accessibility of local marine resources likely also provided a significant buffer to increased terrestrial aridity.

Concordance between OES isotopic shifts and environmental and subsistence records can also be seen at KDS. Enrichment in OES δ^13^C and δ^18^O in layer PBC of the KDS sequence, alongside faunal and shellfish proxy evidence for an increased influence of summer rainfall in the region, is accompanied by increasing densities of shellfish, at a time of increased near-shore upwelling, faunal remains, and diversity of faunal taxa represented in the occupation layers [28] (Fig 5). This may reflect the development of increased dietary and subsistence breadth in response to increasingly arid conditions and a shift in the local rainfall regime. The coastline would also have been consistently further away from KDS (*c*. 15 km) during human occupation than was the case for BBC [10], perhaps suggesting that an increased focus on shellfish, among faunal diversity in general, is an active human subsistence choice in layer PBC. The return to more humid, winter rainfall conditions in layer PAZ is then followed by a shift to more mixed, fynbos, grassy, and rocky faunal indicators [28].

Since Stiner and colleagues’ research into the “Broad Spectrum Revolution” [72–73], dietary breadth has been a popular focus in archaeological studies of human responses to environmental and demographic pressures. However, while traditionally dietary breadth has often been associated with climatic downturns, new models suggest that it may equally be an adaptive solution to productive environments [74]. At BBC, multiple factors appear to stimulate subsistence breadth from Phase M2 onwards. Increased access to marine resources, as a result of closer shorelines, represents favourable conditions for subsistence expansion. By contrast, increasing terrestrial aridity, indicated by palaeoenvironmental proxies at the site, may have led to new technological strategies, including the big-game hunting armatures and the hunting of large mammals, as well as increased exploitation of small game, including tortoises and small mammals. At KDS, a uniform, more distant, coastline implies that increasing use of coastal resources and increased faunal diversity in layer PBC represent a direct response to more unfavourable terrestrial climates. Evidence from these sites confirms that discussions of “dietary breadth” should be locale-specific rather than relying on simple universal models [74].

The cultural and technological innovations of the Still Bay and Howiesons Poort have also often been linked to climatic change. It has been argued that the emergence of material expression and personal ornamentation in the form of ochre processing and engraving, and the manufacture of shell beads, is linked to increasing climatic stress and the necessity of social interaction and exchange [2,7], while the Still Bay and Howiesons Poort technologies have both been linked to new prey and subsistence opportunities brought about by regional climatic and environmental shifts [8,41]. However, at BBC, climatic and environmental variation, as indicated by OES δ^13^C and δ^18^O, does not occur in phase with some of the earliest, and most discussed, material traces of MSA technological and cultural innovation. Fig 5 shows that the processing of ochre began long prior to any climatic or environmental shifts at BBC. Similarly, the bone tools, marine shell beads, engraved ochre, and stone technologies of the Still Bay (now considered to be multi-purpose tools [41]) do not appear in the sequence until a little while *after* the major climatic and environmental change towards increasingly arid conditions and year-round rainfall influence, and after subsistence responses to these changes.

At KDS, the presence of the Howiesons Poort represents a major, early shift in human hunting strategy. At Sibudu Cave, Lombard has argued that Howiesons Poort backed segments were used as transversely backed arrowheads [5] that would have facilitated larger, more dangerous, animals to be captured. However, the Howiesons Poort technology itself remains relatively constant through a period of significant climatic and environmental instability at KDS, indicating its potential contribution to human stability in the face of external environmental change. That said, in layer PBC, with the increased aridity and summer rainfall influence indicated by the OES isotopic data, there is a change in lithic raw material proportions and tool types from the lower layers, including an increase in quartz exploitation and backed and segment tools [28] (Fig 5). The shift in raw materials may be linked to increasing mobility associated with the increased exploitation of large grazing taxa from the emerging grassland biome in this level. Yet, Howiesons Poort toolkits, as well as ochre processing, remain present throughout the environmental variability indicated by the KDS OES sequence.

## Conclusions

### Climatic correlation with early human subsistence, cultural, and technological innovation in southern Africa

Researchers have long sought to link the appearance and/or disappearance of the Still Bay and Howiesons Poort industries to environmental change [7–8,75]. However, although absolute chronologies have improved, studies linking the two are based largely on extrapolating generalizations from non-specific, often off-site, climatic records [13]. This is particularly problematic given Jacobs *et al*.’s [2] observation that Still Bay and Howiesons Poort sites span a number of different biomes across southern Africa, making it unlikely that a given climatic shift can fully explain the entirety of the variance in these technologies.

Previous studies along the southern coast of South Africa have demonstrated that sea-level change likely played an important role in human social, demographic and subsistence responses [9–10]. We have suggested here that, particularly at BBC, proximity to the coast may have influenced subsistence ‘breadth’ and provided an important resource in the face of changing terrestrial environments. While the data are currently less resolved for KDS, future work may clarify the role of sea level in human subsistence strategies at this site. Changing sea levels and bathymetry have also, in part, influenced the preservation of archaeological sites [9]. Indeed, the low numbers of excavated archaeological sites along this coastline containing both Still Bay and Howiesons Poort techno-complexes exacerbates the difficulties of research relating to the periods 77–59ka.

Given the relative scarcity of sites, and their local ecological and cultural variability, it is important to develop records of climatic and environmental change that are closely associated with the archaeological evidence they are hypothesized to explain. The results of one such direct comparison, shown here, urges caution in the construction of broad, generalized models of human climatic response. Stable carbon and oxygen isotope analysis of OES provides a well-understood record of paleoenvironmental change. OES is ubiquitous throughout African archaeological sequences from *c*. 100 ka to the present, while ostriches themselves are highly territorial and therefore represent a local record of environmental conditions. Application of this method to BBC and KDS has revealed that human subsistence responses during the Still Bay and Howiesons Poort were linked to local manifestations of wider climatic changes on the southern Cape coast, including fluctuations in the proportion of grassland and aridity. However, while technological changes may have a more complex, indirect relationship with these environmental changes, we find no evidence that climate *directly* drove the technological or cultural innovations of the Still Bay and Howiesons Poort at the sites of BBC and KDS.

The data presented here thus urges caution in relying on climatic or environmental factors as theoretical drivers of cultural change (contra [8,13]). The palaeoenvironmental proxies from KDS and BBC, as well as wider bathymetric modelling for the region [9], indicate that there was significant variation in the terrestrial and marine resources available to humans at these sites during the periods of occupation. However, while these changes may have impacted human subsistence strategies, they did not directly influence technological or cultural innovation. Indeed, the entirety of early human material cultural florescence associated with the Still Bay and Howiesons Poort traditions in the southern Cape, or southern Africa more widely, cannot be uniformly linked to climatic and environmental forcing. In fact, the data reported here, as suggested elsewhere [10], show that the southern coastal plain of South Africa offered a relatively mesic, stable environment for human technological, cultural and subsistence experimentation (as per Zeder [74]) [2]. It seems that although climatic and environmental change clearly occurred in this region, a diversity of potential resources allowed human populations to absorb these changes.

As a result, although our species has shown itself to be highly resilient in the face of climatic and environmental instability it is clearly not wholly dependent on such changes for its innovation. It may be argued that other causes of human innovation during the MSA of southern Africa may prove more appropriate. Nevertheless, other broad theories, such as cognitive change [76] or demographic drivers [77], face their own issues. Demographic arguments for changes in cultural complexity are coming increasingly under scrutiny, with broad hypotheses not necessarily standing up to on-the-ground testing [78]. As a consequence, we would argue that contextually-appropriate investigations of regional human cultural, technological, and subsistence change are more suitable. Changes in long-distance contact, socio-cultural interactions, population movements, and environmental drivers may all play a role but their impact will be different across the diversity of ecosystems and social networks our species expanded into within Africa during the Late Pleistocene. Whatever their cause, MSA subsistence, cultural, and technological changes in the southern Cape are best seen as the beginning of multi-faceted, flexible material adaptations characteristic of our species.

## Supporting Information

S1 File Supporting Information for: Climate, environment and early human innovation: Stable isotope and faunal proxy evidence from archaeological sites (98-59ka) in the southern Cape, South Africa. (DOCX)

S2 File Full faunal and shellfish specimen lists by excavation quadrat. (XLSX)
