# Supplementary material for: Climate, Environment and Early Human Innovation: Stable Isotope and Faunal Proxy Evidence from Archaeological Sites (98-59ka) in the Southern Cape, South Africa
Source: PLoS One. 2016 Jul 6;11(7):e0157408. doi: 10.1371/journal.pone.0157408 (PMC4934875; doi:10.1371/journal.pone.0157408)
Supplement: S1 File — (DOCX) [file pone.0157408.s001.docx]

**Supporting Information for:**

**Climate, environment and early human innovation: Stable isotope and faunal proxy evidence from archaeological sites (98-59ka) in the southern Cape, South Africa**

Patrick Roberts^1*^, Christopher S. Henshilwood^2,3^, Karen L. van Niekerk^3,2^, Petro Keene^2^, Andrew Gledhill^4^, Jerome Reynard^2,5^, Shaw Badenhorst^6^, Julia Lee-Thorp^1^

^1^School of Archaeology, Research Laboratory for Archaeology and the History of Art, the University of Oxford, Dyson Perrins Building, South Parks Road, Oxford, United Kingdom.

^2^Evolutionary Studies Institute, University of the Witwatersrand, Jan Smuts Avenue, Braamfontein 2000, Johannesburg, South Africa.

^3^Department of Archaeology, History, Cultural Studies and Religion, University of Bergen, Bergen, Norway.

^4^Division of Geographic, Archaeological and Environmental Sciences, University of Bradford, Bradford, United Kingdom.

^5^School of Geography, Archaeology and Environmental Studies, University of the Witwatersrand, Johannesburg, South Africa.

^6^Archaeozoology and Large Mammal Section, Ditsong National Museum of Natural History (former Transvaal Museum), Paul Kruger St, Pretoria, South Africa & Department of Anthropology and Archaeology, University of South Africa, UNISA, South Africa.

*Corresponding author
Email: [patrick.roberts@rlaha.ox.ac.uk](mailto:patrick.roberts@rlaha.ox.ac.uk) (PR)

**This PDF file includes:**

Figures A and B

Tables A to O

Supporting Information Reference List

**Figure A in S1 File.** Plot of OES δ^13^C versus δ^18^O for Blombos Cave.

**Figure B in S1 File.** Plot of OES δ^13^C versus δ^18^O for Klipdrift Shelter.

**Table A in S1 File.** Chronometric information available from Blombos Cave.

| **Phase** | **Layer** | **Sample** | **Age*** | **Uncertainty** | **Method** | **Material** | **Reference** |
| --- | --- | --- | --- | --- | --- | --- | --- |
| BBC HIATUS | DUN | ZB15 | 69.2 | 3.9 | MG-OSL | Quartz | [79] |
|  |  | ZB15 | 67.8 | 4.2 | SG-OSL | Quartz | [51] |
| BBC M1 | WAB | 97253 | 64 (EU, 81 (LU) | 10 (EU), 14( LU) | ESR | Enamel | [40] |
|  | BZB | 97254 | 64 (EU), 87 (LU) | 6 (EU), 11 (LU) | ESR | Enamel | [40] |
|  |  | BB24 | 81 | 10 | TL | Silcrete | [49] |
|  | CA | BB23 | 67 | 7 | TL | Quartzite | [49] |
|  |  | BB20 | 77 | 8 | TL | Silcrete | [49] |
|  |  | 97252 | 63 (EU), 79 (LU) | 8 (EU), 12 (LU) | ESR | Enamel | [40] |
|  |  | 97256 | 57 (EU), 74 (LU) | 7 (EU), 11 (LU) | ESR | Enamel | [40] |
|  | CB | 97257 | 68 (ED), 82 (LU) | 10 (EU), 13 (LU) | ESR | Enamel | [40] |
|  | CC | BB15 | 68 | 6 | TL | Silcrete | [49] |
|  |  | BB12 | 82 | 8 | TL | Silcrete | [49] |
|  |  | 97258 | 70 | 7 (EU), 11 (LU) | ESR | Enamel | [40] |
|  |  | ZB4 | 76 | 4 | MG-OSL | Quartz | [51] |
|  |  | ZB4 | 73 | 3 | MG-OSL | Quartz | [51] |
|  |  | ZB4 | 72.7 | 3.1 | SG-OSL | Quartz | [51] |
|  |  | ZB4 | 72.5 | 4.6 | SG-OSL | Quartz | [29] |
|  |  | BBC10-2 | 74.6 | 3.9 | SG-OSL | Quartz | [29] |
|  | CD | 97259 | 50 (EU), 70 (LU) | 5 (EU), 9 (LU) | ESR | Enamel | [40] |
|  |  | BBC10-3 | 74.9 | 4.3 | SG-OSL | Quartz | [29] |
| BBC M2 UPPER | CFA | BBC10-4 | 69.7 | 3.9 | SG-OSL | Quartz | [29] |
|  | CFB/CFC | BB6 | 105 | 9 | TL | Silcrete | [49] |
|  |  | BB10-5 | 75.5 | 5 | SG-OSL | Quartz | [29] |
|  |  | BB10-6 | 68.8 | 4.8 | SG-OSL | Quartz | [29] |
|  | CFD | ZB10 | 81 | 4 | MG-OSL | Quartz | [51] |
|  |  | ZB10 | 79 | 3 | MG-OSL | Quartz | [51] |
|  |  | ZB10 | 76.8 | 3.1 | SG-OSL | Quartz | [51] |
|  |  | ZB10 | 76.7 | 4.8 | SG-OSL | Quartz | [29] |
| BBC M2 LOWER | CGAA | ZB7 | 75 | 2 | MG-OSL | Quartz | [51] |
|  |  | ZB7 | 79 | 4 | MG-OSL | Quartz | [51] |
|  |  | ZB7 | 82.2 | 3.6 | SG-OSL | Quartz | [51] |
|  |  | BBC10-7 | 78.8 | 5.6 | SG-OSL | Quartz | [29] |
|  |  | BBC10-8 | 78.9 | 5.9 | SG-OSL | Quartz | [29] |
|  | CGAB |  |  |  |  |  |  |
|  | CGAC | BB1 | 76 | 7 | TL | Quartzite | [49] |
|  |  | ZB8 | 77 | 4 | MG-OSL | Quartz | [51] |
|  |  | ZB8 | 79 | 4 | MG-OSL | Quartz | [51] |
|  |  | ZB8 | 81.1 | 4.2 | SG-OSL | Quartz | [51] |
|  |  | ZB6 | 81 | 4 | MG-OSL | Quartz | [51] |
|  |  | ZB6 | 88 | 5 | MG-OSL | Quartz | [51] |
|  |  | ZB6 | 84.6 | 5.8 | SG-OSL | Quartz | [51] |
| BBC M3 | CH/CI | ZB42 | 98.9 | 4.5 | MG-OSL | Quartz | [51] |
|  |  | BBC08-10 | 96 | 6 | SG-OSL | Quartz | [39] |
|  |  | BBC08-4 | 89 | 6 | SG-OSL | Quartz | [39] |
|  |  | BBC08-12 | 91 | 6 | SG-OSL | Quartz | [39] |
|  |  | BBC08-11 | 100 | 6 | SG-OSL | Quartz | [39] |
|  | CJ | ZB5 | 143.2 | 5.5 | MG-OSL | Quartz | [39] |
|  |  | ZB5 | 97 | 5 | SG-OSL | Quartz | [39] |
|  | CJh1 | BBC08-3 | 97 | 6 | SG-OSL | Quartz | [39] |
|  | CK |  |  |  |  |  |  |
|  | CL |  |  |  |  |  |  |
|  | CM |  |  |  |  |  |  |
|  | CN/CO |  |  |  |  |  |  |
|  | CP | BBC08-6 | 98 | 6 | SG-OSL |  | [39] |
|  |  | BBC08-2 | 100 | 7 | SG-OSL | Quartz | [39] |
|  |  | BBC08-5 | 100 | 7 | SG-OSL | Quartz | [39] |
|  |  | BBC08-1 | 107 | 7 | SG-OSL | Quartz | [39] |
|  |  |  | >92 |  | Th/U | Calcite | [39] |

*Where the same samples were dated more than once by the same author using the same technique, the most recently published date is considered to be the most accurate and is given in table S2.

**Table B in S1 File.** Single-grain OSL dates available from Blombos Cave and Klipdrift Shelter.

| **Site** | **Sample** | **Phase** | **Layer** | **Age (ka)*** | **Uncertainty (± ka)** | **Reference** |
| --- | --- | --- | --- | --- | --- | --- |
| **Klipdrift Shelter** | DS3 | HP | PAY | 60 | 4 | [28] |
| **Klipdrift Shelter** | DS2 | HP | PBA/PBB | 59.4 | 4.6 | [28] |
| **Klipdrift Shelter** | DS1 | HP | PBC | 65.5 | 4.8 | [28] |
| **Klipdrift Shelter** | DS9 | HP | PBD | 64.6 | 4.2 | [28] |
| **Klipdrift Shelter** | DS10 | HP | PCA | 63.5 | 4.7 | [28] |
| **Klipdrift Shelter** | DS7 | HP | PE | 71.6 | 5.1 | [28] |
| **Blombos Cave** | ZB15 | BBC HIATUS | HIATUS | 67.8 | 4.2 | [51] |
| **Blombos Cave** | BBC10-2 | BBC M1 | CC | 74.6 | 3.9 | [29] |
| **Blombos Cave** | ZB4 | BBC M1 | CC | 72.5 | 4.6 | [39] |
| **Blombos Cave** | BBC10-3 |  | CD | 74.9 | 4.3 | [39] |
| **Blombos Cave** | BBC10-4 | BBC M2 UPPER | CFA | 69.7 | 3.9 | [39] |
| **Blombos Cave** | BBC10-5 | BBC M2 UPPER | CFB/CFC | 75.5 | 5 | [39] |
| **Blombos Cave** | BBC10-6 | BBC M2 UPPER | CFB/CFC | 68.8 | 4.6 | [39] |
| **Blombos Cave** | ZB10 | BBC M2 UPPER | CFD | 76.7 | 4.8 | [39] |
| **Blombos Cave** | ZB7 | BBC M2 LOWER | CGAA | 82.2 | 3.6 | [39] |
| **Blombos Cave** | BBC10-7 | BBC M2 LOWER | CGAA | 78.8 | 5.6 | [39] |
| **Blombos Cave** | BBC10-8 | BBC M2 LOWER | CGAA | 78.9 | 5.9 | [39] |
| **Blombos Cave** | ZB8 | BBC M2 LOWER | CGAC | 81.1 | 4.4 | [51] |
| **Blombos Cave** | ZB6 | BBC M2 LOWER | CGAC | 84.6 | 5.8 | [51] |
| **Blombos Cave** | BBC08-10 |  | CH | 96 | 6 | [39] |
| **Blombos Cave** | BBC08-4 |  | CIB | 89 | 6 | [39] |
| **Blombos Cave** | BBC08-12 |  | CIBh1 | 91 | 6 | [39] |
| **Blombos Cave** | BBC08-11 |  | CIB | 100 | 6 | [39] |
| **Blombos Cave** | ZB5 |  | CJ | 97 | 5 | [39] |
| **Blombos Cave** | BBC08-3 |  | CJh1 | 97 | 6 | [39] |
| **Blombos Cave** | BBC08-6 |  | CP Upper | 98 | 6 | [39] |
| **Blombos Cave** | BBC08-2 |  | CP/CPA | 100 | 7 | [39] |
| **Blombos Cave** | BBC08-5 |  | CP/CPA | 100 | 7 | [39] |
| **Blombos Cave** | BBC08-1 |  | CP/CPA | 106 | 7 | [39] |

*****Where the same samples were dated more than once by the same author using the same technique, the most recently published date is considered to be the most accurate and is given in table S2.

**Table C in S1 File.** List of OES samples from Blombos Cave.

| **Layer** | **Quadrat** | **Date excavated** | **Number of samples** | **Sample** |
| --- | --- | --- | --- | --- |
| CC | D5 | 15.02.2008 | 5 | BLOMB 1 |
|  |  |  |  | BLOMB 2 |
|  |  |  |  | BLOMB 3 |
|  |  |  |  | BLOMB 4 |
|  |  |  |  | BLOMB 5 |
|  | F7b | 29.11.11 | 2 | BLOMB 6 |
|  |  |  |  | BLOMB 7 |
|  | G7a | 08.12.11 | 4 | BLOMB 8 |
|  |  |  |  | BLOMB 9 |
|  |  |  |  | BLOMB 10 |
|  |  |  |  | BLOMB 11 |
| CD | F6d | 15.04.2010 | 4 | BLOMB 12 |
|  |  |  |  | BLOMB 13 |
|  |  |  |  | BLOMB 14 |
|  |  |  |  | BLOMB 15 |
|  | G6c | 14.04.2010 | 1 | BLOMB 16 |
|  | F7b | 26.11.13 | 2 | BLOMB 17 |
|  |  |  |  | BLOMB 18 |
| CF | F6d | 22.04.2010 | 1 | BLOMB 19 |
|  | G6c | 21.04.2010 | 3 | BLOMB 20 |
|  |  |  |  | BLOMB 21 |
|  |  |  |  | BLOMB 22 |
|  | H6c | 13.04.2010 | 1 | BLOMB 23 |
|  | F7b | 02.12.13 | 2 | BLOMB 24 |
|  |  |  |  | BLOMB 25 |
|  | H7a | 06.12.11 | 2 | BLOMB 26 |
|  |  |  |  | BLOMB 27 |
|  | G6d | 12.04.2010 | 2 | BLOMB 28 |
|  |  |  |  | BLOMB 29 |
| CI | H6c | 01.11.2011 | 1 | BLOMB 30 |
|  | H6c | 04.11.2011 | 1 | BLOMB 31 |
|  | E5c | 04.03.2008 | 1 | BLOMB 32 |
|  | G6c | 22.11.2011 | 2 | BLOMB 33 |
|  |  |  |  | BLOMB 34 |
|  | H6d | 22.11.2011 | 1 | BLOMB 35 |
|  | E5d | 27.02.08 | 1 | BLOMB 36 |
|  | E5c | 03.03.08 | 2 | BLOMB 37 |
|  |  |  |  | BLOMB 38 |
|  | E6b | 10.03.08 | 1 | BLOMB 39 |
| CJ | H2 F5c | 24/28.05.2007 | 3 | BLOMB 40 |
|  |  |  |  | BLOMB 41 |
|  |  |  |  | BLOMB 42 |
|  | CJ hiatus F6a | 17.05.2007 | 2 | BLOMB 43 |
|  |  |  |  | BLOMB 44 |
|  | CJ hiatus F5c | 24.05.2007 | 2 | BLOMB 45 |
|  |  |  |  | BLOMB 46 |
|  | CJ hiatus F5d | 31.05.2007 | 1 | BLOMB 47 |
|  | CJ hiatus E6b | 24.03.2009 | 1 | BLOMB 48 |
| CK | F5c | 01/05.06.2007 | 3 | BLOMB 49 |
|  |  |  |  | BLOMB 50 |
|  |  |  |  | BLOMB 51 |
|  | F5d | 05.06.2007 | 3 | BLOMB 52 |
|  |  |  |  | BLOMB 53 |
|  |  |  |  | BLOMB 54 |
|  | F5d | 01.06.2001 | 3 | BLOMB 55 |
|  |  |  |  | BLOMB 56 |
|  |  |  |  | BLOMB 57 |
| CL | F5d | 05.06.2007 | 2 | BLOMB 58 |
|  |  |  |  | BLOMB 59 |
|  | F6a | 06.06.2007 | 1 | BLOMB 60 |
|  | G5c | 07.05.2007 | 2 | BLOMB 61 |
|  |  |  |  | BLOMB 62 |
|  | G5c | 07.05.2007 | 5 | BLOMB 63 |
|  |  |  |  | BLOMB 64 |
|  |  |  |  | BLOMB 65 |
|  |  |  |  | BLOMB 66 |
|  |  |  |  | BLOMB 67 |
| CN/CO | F6d | 29.11.2011 | 3 | BLOMB 68 |
|  |  |  |  | BLOMB 69 |
|  |  |  |  | BLOMB 70 |
|  | G6c | 28.11.2011 | 2 | BLOMB 71 |
|  |  |  |  | BLOMB 72 |
|  |  |  |  | BLOMB 73 |
|  |  |  |  | BLOMB 74 |
|  |  |  |  | BLOMB 75 |
|  |  |  |  | BLOMB 76 |
|  |  |  |  | BLOMB 77 |
|  | H5c | 11.05.2007 | 1 | BLOMB 78 |
|  | H6b | 18.02.2008 | 1 | BLOMB 79 |
|  | F6d | 29.11.2011 | 6 | BLOMB 80 |
|  |  |  |  | BLOMB 81 |
|  |  |  |  | BLOMB 82 |
|  |  |  |  | BLOMB 83 |
|  |  |  |  | BLOMB 84 |
|  |  |  |  | BLOMB 85 |
|  | G6c | 28.11.11 | 5 | BLOMB 86 |
|  |  |  |  | BLOMB 87 |
|  |  |  |  | BLOMB 88 |
|  |  |  |  | BLOMB 89 |
|  |  |  |  | BLOMB 90 |

| **Layer** | **Quadrat** | **Date excavated** | **Number of samples** | **Sample** |
| --- | --- | --- | --- | --- |
| PAZ | R29d | 03.03.2011 | 1 | KDS 1 |
|  | R29c | 07.03.2011 | 1 | KDS 2 |
|  |  |  |  | KDS 3 |
|  |  |  |  | KDS 4 |
|  | R30c | 03.03.2011 | 3 | KDS 5 |
|  |  |  |  | KDS 6 |
|  |  |  |  | KDS 7 |
|  | R30b | 24.02.2012 | 1 | KDS 8 |
| PBA/PBB | R30c | 09.03.2011 | 5 | KDS 9 |
|  |  |  |  | KDS 10 |
|  |  |  |  | KDS 11 |
|  |  |  |  | KDS 12 |
|  |  |  |  | KDS 13 |
|  | R319 | 26.02.2013 | 2 | KDS 14 |
|  |  |  |  | KDS 15 |
| PBC | R30a | 07.03.2013 | 5 | KDS 16 |
|  |  |  |  | KDS 17 |
|  |  |  |  | KDS 18 |
|  |  |  |  | KDS 19 |
|  |  |  |  | KDS 20 |
|  | S29b | 22.02.2011 | 3 | KDS 21 |
|  |  |  |  | KDS 22 |
|  |  |  |  | KDS 23 |
|  | R29d | 04.03.2011 | 3 | KDS 24 |
|  |  |  |  | KDS 25 |
|  |  |  |  | KDS 26 |
| PCA | S29a | 03.03.2011 | 3 | KDS 27 |
|  |  |  |  | KDS 28 |
|  |  |  |  | KDS 29 |
|  | S29b | 24.02.2011 | 1 | KDS 30 |
|  | R28d | 02.03.2012 | 1 | KDS 31 |
|  | R28d | 06.03.2012 | 1 | KDS 32 |
|  | R28c | 02.03.2012 | 1 | KDS 33 |
|  | R30d | 23.02.2012 | 1 | KDS 34 |
| PDA | S29d | 03.03.2011 | 5 | KDS 35 |
|  |  |  |  | KDS 36 |
|  |  |  |  | KDS 37 |
|  |  |  |  | KDS 38 |
|  |  |  |  | KDS 39 |
|  | S29b | 24.02.2011 | 1 | KDS 40 |
|  | S29d | 03.03.2011 | 2 | KDS 41 |
|  |  |  |  | KDS 42 |

**Table D in S1 File**. List of OES samples from Klipdrift Shelter.

**Table E in S1 File.** OES δ^13^C and δ^18^O measurements from Blombos Cave.

| **Layer** | **Quadrat** | **Date excavated** | **Number of samples** | **Sample** | **δ^13^C(**‰**) (VPDB)** | **δ^18^O(**‰**) (VSMOW)** |
| --- | --- | --- | --- | --- | --- | --- |
| CC | D5 | 15.02.2008 | 5 | BLOMB 1 | -9.6 | 32.9 |
|  |  |  |  | BLOMB 2 | -10.4 | 31.7 |
|  |  |  |  | BLOMB 3 | -9.4 | 32.8 |
|  |  |  |  | BLOMB 4 | -9.9 | 34.4 |
|  |  |  |  | BLOMB 5 | -8.3 | 34.8 |
|  | F7b | 29.11.11 | 2 | BLOMB 6 | -9.5 | 33.5 |
|  |  |  |  | BLOMB 7 | -9.9 | 29.9 |
|  | G7a | 08.12.11 | 4 | BLOMB 8 | -8.8 | 33.0 |
|  |  |  |  | BLOMB 9 | -8.0 | 35.4 |
|  |  |  |  | BLOMB 10 | -9.8 | 35.0 |
|  |  |  |  | BLOMB 11 | -9.7 | 33.3 |
| CD | F6d | 15.04.2010 | 4 | BLOMB 12 | -8.7 | 36.0 |
|  |  |  |  | BLOMB 13 | -10.1 | 31.8 |
|  |  |  |  | BLOMB 14 | -8.4 | 36.3 |
|  |  |  |  | BLOMB 15 | -9.6 | 31.5 |
|  | G6c | 14.04.2010 | 1 | BLOMB 16 | -10.2 | 31.1 |
|  | F7b | 26.11.2013 | 2 | BLOMB 17 | -9.1 | 34.9 |
|  |  |  |  | BLOMB 18 | -9.5 | 32.8 |
| CF | F6d | 22.04.2010 | 1 | BLOMB 19 | -10.3 | 32.7 |
|  | G6c | 21.04.2010 | 3 | BLOMB 20 | -9.7 | 31.9 |
|  |  |  |  | BLOMB 21 | -10.5 | 33.1 |
|  |  |  |  | BLOMB 22 | -10.3 | 32.6 |
|  | H6c | 13.04.2010 | 1 | BLOMB 23 | -9.1 | 34.4 |
|  | F7b | 02.12.13 | 2 | BLOMB 24 | -10.0 | 32.3 |
|  |  |  |  | BLOMB 25 | -8.7 | 32.6 |
|  | H7a | 06.12.11 | 2 | BLOMB 26 | -9.1 | 31.9 |
|  |  |  |  | BLOMB 27 | -8.8 | 33.7 |
|  | G6d | 12.04.2010 | 2 | BLOMB 28 | -9.0 | 33.5 |
|  |  |  |  | BLOMB 29 | -10.5 | 31.7 |
| CI | H6c | 01.11.2011 | 1 | BLOMB 30 | -9.2 | 31.8 |
|  | H6c | 04.11.2011 | 1 | BLOMB 31 | -9.1 | 31.9 |
|  | E5c | 04.03.2008 | 1 | BLOMB 32 | -9.8 | 33.4 |
|  | G6c | 22.11.2011 | 2 | BLOMB 33 | -9.9 | 33.2 |
|  |  |  |  | BLOMB 34 | -9.8 | 33.6 |
|  | H6d | 22.11.2011 | 1 | BLOMB 35 | -9.3 | 31.7 |
|  | E5d | 27.02.08 | 1 | BLOMB 36 | -9.4 | 31.3 |
|  | E5c | 03.03.08 | 2 | BLOMB 37 | -9.4 | 31.3 |
|  |  |  |  | BLOMB 38 | -8.7 | 32.5 |
|  | E6b | 10.03.08 | 1 | BLOMB 39 | -9.1 | 33.3 |
| CJ | H2 F5c | 24/28.05.2007 | 3 | BLOMB 40 | -8.6 | 31.7 |
|  |  |  |  | BLOMB 41 | -12.1 | 31.4 |
|  |  |  |  | BLOMB 42 | -10.8 | 33.1 |
|  | CJ hiatus F6a | 17.05.2007 | 2 | BLOMB 43 | -12.1 | 31.5 |
|  |  |  |  | BLOMB 44 | -12.0 | 31.6 |
|  | CJ hiatus F5c | 24.05.2007 | 2 | BLOMB 45 | -8.3 | 31.9 |
|  |  |  |  | BLOMB 46 | -10.2 | 29.7 |
|  | CJ hiatus F5d | 31.05.2007 | 1 | BLOMB 47 | -8.1 | 34.0 |
|  | CJ hiatus E6b | 24.03.2009 | 1 | BLOMB 48 | -8.5 | 31.4 |
| CK | F5c | 01/05.06.2007 | 3 | BLOMB 49 | -12.2 | 30.1 |
|  |  |  |  | BLOMB 50 | -9.7 | 36.3 |
|  |  |  |  | BLOMB 51 | -10.5 | 29.7 |
|  | F5d | 05.06.2007 | 3 | BLOMB 52 | -10.5 | 33.2 |
|  |  |  |  | BLOMB 53 | -10.5 | 33.0 |
|  |  |  |  | BLOMB 54 | -10.2 | 33.0 |
|  | F5d | 01.06.2001 | 3 | BLOMB 55 | -10.6 | 29.2 |
|  |  |  |  | BLOMB 56 | -10.0 | 33.0 |
|  |  |  |  | BLOMB 57 | -11.5 | 30.9 |
| CL | F5d | 05.06.2007 | 2 | BLOMB 58 | -10.3 | 29.6 |
|  |  |  |  | BLOMB 59 | -12.2 | 31.6 |
|  | F6a | 06.06.2007 | 1 | BLOMB 60 | -10.8 | 29.8 |
|  | G5c | 07.05.2007 | 2 | BLOMB 61 | -11.5 | 30.4 |
|  |  |  |  | BLOMB 62 | -10.2 | 29.9 |
|  | G5c | 07.05.2007 | 5 | BLOMB 63 | -10.0 | 29.5 |
|  |  |  |  | BLOMB 64 | -10.5 | 29.4 |
|  |  |  |  | BLOMB 65 | -10.7 | 29.5 |
|  |  |  |  | BLOMB 66 | -10.6 | 33.3 |
|  |  |  |  | BLOMB 67 | -12.1 | 30.8 |
| CN/CO | F6d | 29.11.2011 | 9 | BLOMB 68 | -12.2 | 30.8 |
|  |  |  |  | BLOMB 69 | -10.2 | 30.9 |
|  |  |  |  | BLOMB 70 | -9.8 | 30.4 |
|  |  |  |  | BLOMB 71 | -9.2 | 35.5 |
|  |  |  |  | BLOMB 72 | -12.1 | 30.8 |
|  |  |  |  | BLOMB 73 | -10.1 | 30.5 |
|  |  |  |  | BLOMB 74 | -9.4 | 31.0 |
|  |  |  |  | BLOMB 75 | -9.1 | 35.9 |
|  |  |  |  | BLOMB 76 | -11.8 | 31.2 |
|  | G6c | 28.11.2011 | 7 | BLOMB 77 | -11.0 | 30.1 |
|  |  |  |  | BLOMB 78 | -9.9 | 30.2 |
|  |  |  |  | BLOMB 79 | -8.8 | 30.5 |
|  |  |  |  | BLOMB 80 | -9.2 | 35.5 |
|  |  |  |  | BLOMB 81 | -9.8 | 31.2 |
|  |  |  |  | BLOMB 82 | -10.5 | 32.5 |
|  |  |  |  | BLOMB 83 | -9.3 | 36.3 |

| **Layer** | **Quadrat** | **Date excavated** | **Number of samples** | **Sample** | **δ^13^C(**‰**) (VPDB)** | **δ^18^O(**‰**) (VSMOW)** |
| --- | --- | --- | --- | --- | --- | --- |
| PAZ | R29d | 03.03.2011 | 1 | KDS 1 | -10.0 | 32.3 |
|  | R29c | 07.03.2011 | 1 | KDS 2 | -11.0 | 29.5 |
|  |  |  |  | KDS 3 | -9.4 | 32.5 |
|  |  |  |  | KDS 4 | -10.0 | 32.8 |
|  | R30c | 03.03.2011 | 3 | KDS 5 | -9.6 | 35.2 |
|  |  |  |  | KDS 6 | -11.2 | 29.4 |
|  |  |  |  | KDS 7 | -9.8 | 32.7 |
|  | R30b | 24.02.2012 | 1 | KDS 8 | -9.6 | 32.9 |
| PBA/PBB | R30c | 09.03.2011 | 5 | KDS 9 | -9.5 | 34.7 |
|  |  |  |  | KDS 10 | -9.8 | 33.0 |
|  |  |  |  | KDS 11 | -8.5 | 35.9 |
|  |  |  |  | KDS 12 | -9.5 | 33.3 |
|  |  |  |  | KDS 13 | -9.2 | 33.0 |
|  | R319 | 26.02.2013 | 2 | KDS 14 | -8.8 | 32.7 |
|  |  |  |  | KDS 15 | -8.3 | 34.2 |
| PBC | R30a | 07.03.2013 | 5 | KDS 16 | -9.1 | 33.4 |
|  |  |  |  | KDS 17 | -8.5 | 36.1 |
|  |  |  |  | KDS 18 | -8.6 | 36.3 |
|  |  |  |  | KDS 19 | -8.3 | 35.9 |
|  |  |  |  | KDS 20 | -8.4 | 36.1 |
|  | S29b | 22.02.2011 | 3 | KDS 21 | -8.2 | 35.4 |
|  |  |  |  | KDS 22 | -8.6 | 35.1 |
|  |  |  |  | KDS 23 | -8.3 | 35.2 |
|  | R29d | 04.03.2011 | 3 | KDS 24 | -8.7 | 33.1 |
|  |  |  |  | KDS 25 | -8.7 | 34.4 |
|  |  |  |  | KDS 26 | -9.0 | 30.7 |
| PCA | S29a | 03.03.2011 | 3 | KDS 27 | -10.1 | 32.0 |
|  |  |  |  | KDS 28 | -10.4 | 31.9 |
|  |  |  |  | KDS 29 | -10.5 | 32.5 |
|  | S29b | 24.02.2011 | 1 | KDS 30 | -9.6 | 36.2 |
|  | R28d | 02.03.2012 | 1 | KDS 31 | -9.5 | 31.6 |
|  | R28d | 06.03.2012 | 1 | KDS 32 | -9.1 | 32.7 |
|  | R28c | 02.03.2012 | 1 | KDS 33 | -9.4 | 35.7 |
|  | R30d | 23.02.2012 | 1 | KDS 34 | -9.3 | 32.4 |
| PDA | S29d | 03.03.2011 | 5 | KDS 35 | -9.2 | 34.5 |
|  |  |  |  | KDS 36 | -9.2 | 34.4 |
|  |  |  |  | KDS 37 | -9.1 | 34.6 |
|  |  |  |  | KDS 38 | -9.2 | 34.7 |
|  |  |  |  | KDS 39 | -9.2 | 34.6 |
|  | S29b | 24.02.2011 | 1 | KDS 40 | -9.7 | 33.3 |
|  | S29d | 03.03.2011 | 2 | KDS 41 | -8.6 | 34.1 |
|  |  |  |  | KDS 42 | -8.8 | 34.0 |

**Table F in S1 File.** OES δ^13^C and δ^18^O measurements from Klipdrift Shelter.

**Table G in S1 File.** Results of ANOVA statistical test and post-hoc Tukey pairwise comparison of site group δ^13^C variance.

|  | **Degrees of freedom** | **Sum of squares** | **Mean squares** | **F-value** | **Pr(>F)** |
| --- | --- | --- | --- | --- | --- |
| Site | 2 | 31.65 | 15.827 | 20.75 | 0.000 |
| Residuals | 122 | 93.07 | 0.763 |  |  |
| **Site** | **Difference** | **Lower** | **Upper** | **Probability adjacent** |  |
| BLOMB2-BLOMB1 | -0.951 | -1.407 | -0.495 | 0.000 |  |
| KDS-BLOMB1 | 0.178 | -0.283 | 0.638 | 0.633 |  |
| KDS-BLOMB2 | 1.129 | 0.681 | 1.576 | 0.000 |  |

**Table H in S1 File.** Results of ANOVA statistical test and post-hoc Tukey pairwise comparison of site group δ^18^O variance.

|  | **Degrees of freedom** | **Sum of squares** | **Mean squares** | **F-value** | **Pr(>F)** |
| --- | --- | --- | --- | --- | --- |
| Site | 2 | 93.9 | 46.95 | 15.76 | 0.000 |
| Residuals | 122 | 363.4 | 2.98 |  |  |
| **Site** | **Difference** | **Lower** | **Upper** | **Probability adjacent** |  |
| BLOMB2-BLOMB1 | -1.330 | -2.230 | -0.429 | 0.003 |  |
| KDS-BLOMB1 | 0.729 | -0.182 | 1.640 | 0.141 |  |
| KDS-BLOMB2 | 2.059 | 1.175 | 2.942 | 0.000 |  |

**Table I in S1 File. Results of ANOVA statistical test and post-hoc Tukey pairwise comparison of layer δ^13^C variance at Blombos Cave.**

|  | **Degrees of freedom** | **Sum of squares** | **Mean squares** | **F-value** | **Pr(>F)** |
| --- | --- | --- | --- | --- | --- |
| Site | 7 | 24.05 | 3.436 | 3.866 | 0.001 |
| Residuals | 75 | 66.65 | 0.889 |  |  |
| **Site** | **Difference** | **Lower** | **Upper** | **Probability adjacent** |  |
| CD-CC | 0.019 | -1.402 | 1.441 | 1.000 |  |
| CF-CC | -0.245 | -1.499 | 1.008 | 0.999 |  |
| CI-CC | 0.021 | -1.263 | 1.305 | 1.000 |  |
| CJ-CC | -0.687 | -2.008 | 0.634 | 0.736 |  |
| CK-CC | -1.242 | -2.564 | 0.079 | 0.080 |  |
| CL-CC | -1.499 | -2.783 | -0.215 | 0.011 |  |
| CN/CO-CC | -0.759 | -1.910 | 0.392 | 0.453 |  |
| CF-CD/CE | -0.265 | -1.686 | 1.156 | 0.999 |  |
| CI-CD | 0.001 | -1.447 | 1.450 | 1.000 |  |
| CJ-CD | -0.706 | -2.188 | 0.775 | 0.812 |  |
| CK-CD | -1.262 | -2.743 | 0.219 | 0.153 |  |
| CL-CD | -1.519 | -2.967 | -0.070 | 0.033 |  |
| CN/CO-CD | -0.779 | -2.111 | 0.554 | 0.607 |  |
| CI-CF | 0.266 | -1.018 | 1.551 | 0.998 |  |
| CJ-CF | -0.441 | -1.763 | 0.880 | 0.966 |  |
| CK-CF | -0.997 | -2.318 | 0.324 | 0.280 |  |
| CL-CF | -1.254 | -2.538 | 0.031 | 0.061 |  |
| CN/CO-CF | -0.514 | -1.665 | 0.638 | 0.859 |  |
| CJ-CI | -0.708 | -2.058 | 0.643 | 0.728 |  |
| CK-CI | -1.263 | -2.614 | 0.087 | 0.084 |  |
| CL-CI | -1.520 | -2.835 | -0.205 | 0.012 |  |
| CN/CO-CI | -0.780 | -1.965 | 0.405 | 0.455 |  |
| CK-CJ | -0.556 | -1.941 | 0.830 | 0.914 |  |
| CL-CJ | -0.812 | -2.163 | 0.538 | 0.572 |  |
| CN/CO-CJ | -0.072 | -1.297 | 1.153 | 1.000 |  |
| CL-CK | -0.257 | -1.607 | 1.094 | 0.999 |  |
| CN/CO-CK | 0.483 | -0.741 | 1.708 | 0.920 |  |
| CN/CO-CL | 0.740 | -0.445 | 1.925 | 0.524 |  |

**Table J in S1 File.** Results of ANOVA statistical test and post-hoc Tukey pairwise comparison of layer δ^18^O variance at Blombos Cave.

|  | **Degrees of freedom** | **Sum of squares** | **Mean squares** | **F-value** | **Pr(>F)** |
| --- | --- | --- | --- | --- | --- |
| Site | 7 | 64.33 | 9.190 | 3.223 | 0.005 |
| Residuals | 75 | 213.89 | 2.852 |  |  |
| **Site** | **Difference** | **Lower** | **Upper** | **Probability adjacent** |  |
| CD-CC | 0.149 | -2.397 | 2.695 | 1.000 |  |
| CF-CC | -0.573 | -2.818 | 1.673 | 0.993 |  |
| CI-CC | -0.936 | -3.237 | 1.364 | 0.907 |  |
| CJ-CC | -1.525 | -3.892 | 0.842 | 0.483 |  |
| CK-CC | -1.292 | -3.659 | 1.075 | 0.686 |  |
| CL-CC | -2.956 | -5.257 | -0.656 | 0.003 |  |
| CN/CO-CC | -1.255 | -3.318 | 0.807 | 0.557 |  |
| CF-CD | -0.722 | -3.268 | 1.824 | 0.987 |  |
| CI-CD | -1.086 | -3.681 | 1.509 | 0.894 |  |
| CJ-CD | -1.675 | -4.328 | 0.979 | 0.510 |  |
| CK-CD | -1.441 | -4.095 | 1.212 | 0.691 |  |
| CL-CD | -3.106 | -5.701 | -0.511 | 0.008 |  |
| CN/CO-CD | -1.404 | -3.791 | 0.982 | 0.599 |  |
| CI-CF | -0.364 | -2.664 | 1.937 | 1.000 |  |
| CJ-CF | -0.953 | -3.319 | 1.414 | 0.912 |  |
| CK-CF | -0.719 | -3.086 | 1.648 | 0.980 |  |
| CL-CF | -2.384 | -4.684 | -0.083 | 0.037 |  |
| CN/CO-CF | -0.682 | -2.745 | 1.380 | 0.968 |  |
| CJ-CI | -0.589 | -3.008 | 1.831 | 0.995 |  |
| CK-CI | -0.356 | -2.775 | 2.064 | 1.000 |  |
| CL-CI | -2.020 | -4.375 | 0.335 | 0.147 |  |
| CN/CO-CI | -0.319 | -2.441 | 1.804 | 1.000 |  |
| CK-CJ | 0.233 | -2.249 | 2.716 | 1.000 |  |
| CL-CJ | -1.431 | -3.851 | 0.988 | 0.592 |  |
| CN/CO-CJ | 0.270 | -1.924 | 2.464 | 1.000 |  |
| CL-CK | -1.664 | -4.084 | 0.755 | 0.397 |  |
| CN/CO-CK | 0.037 | -2.157 | 2.231 | 1.000 |  |
| CN/CO-CL | 1.701 | -0.421 | 3.824 | 0.212 |  |

**Table K in S1 File.** Results of ANOVA statistical test and post-hoc Tukey pairwise comparison of layer δ^13^C variance at Klipdrift Shelter.

|  | **Degrees of freedom** | **Sum of squares** | **Mean squares** | **F-value** | **Pr(>F)** |
| --- | --- | --- | --- | --- | --- |
| Site | 4 | 12.55 | 3.137 | 13.63 | 0.000 |
| Residuals | 37 | 8.51 | 0.230 |  |  |
| **Site** | **Difference** | **Lower** | **Upper** | **Probability adjacent** |  |
| PBA/PBB-PAZ | 0.989 | 0.278 | 1.701 | 0.003 |  |
| PBC-PAZ | 1.493 | 0.854 | 2.132 | 0.000 |  |
| PCA-PAZ | 0.338 | -0.350 | 1.025 | 0.627 |  |
| PDA-PAZ | 0.950 | 0.262 | 1.638 | 0.003 |  |
| PBC-PBA/PBB | 0.504 | -0.161 | 1.169 | 0.213 |  |
| PCA-PBA/PBB | -0.652 | -1.364 | 0.060 | 0.086 |  |
| PDA-PBA/PBB | -0.039 | -0.751 | 0.672 | 1.000 |  |
| PCA-PBC | -1.156 | -1.795 | -0.517 | 0.000 |  |
| PDA-PBC | -0.543 | -1.182 | 0.096 | 0.128 |  |
| PDA-PCA | 0.613 | -0.075 | 1.300 | 0.101 |  |

**Table L in S1 File.** Results of ANOVA statistical test and post-hoc Tukey pairwise comparison of layer δ^18^O variance at Klipdrift Shelter.

|  | **Degrees of freedom** | **Sum of squares** | **Mean squares** | **F-value** | **Pr(>F)** |
| --- | --- | --- | --- | --- | --- |
| Site | 4 | 35.31 | 8.828 | 3.781 | 0.011 |
| Residuals | 37 | 86.38 | 2.335 |  |  |
| **Site** | **Difference** | **Lower** | **Upper** | **Probability adjacent** |  |
| PBA/PBB-PAZ | 1.666 | -0.601 | 3.933 | 0.239 |  |
| PBC-PAZ | 2.538 | 0.502 | 4.573 | 0.008 |  |
| PCA-PAZ | 0.963 | -1.228 | 3.153 | 0.717 |  |
| PDA-PAZ | 2.113 | -0.078 | 4.303 | 0.063 |  |
| PBC-PBA/PBB | 0.871 | -1.246 | 2.989 | 0.763 |  |
| PCA-PBA/PBB | -0.704 | -2.971 | 1.564 | 0.899 |  |
| PDA-PBA/PBB | 0.446 | -1.821 | 2.714 | 0.979 |  |
| PCA-PBC | -1.575 | -3.610 | 0.460 | 0.196 |  |
| PDA-PBC | -0.425 | -2.460 | 1.610 | 0.974 |  |
| PDA-PCA | 1.150 | -1.040 | 3.340 | 0.566 |  |

| **Diet** | **Taxa** | **Common name** | | **CA** | **CB** | **CC** | **CD** | **CF** | **M1 (KC*)** | | **M2 (KC*)** |
| --- | --- | --- | --- | --- | --- | --- | --- | --- | --- | --- | --- |
| br | *Diceros bicornis* | Black rhinoceros | | - | - | - | - | - | 3 | - | |
| gr | *Equus* sp*.* | Zebra | | 1 | - | - | - | - | - | - | |
| gr | *Redunca arundinum* | Southern reedbuck | | - | - | - | - | - | 14 | 4 | |
| gr | *Redunca* sp. | Reedbuck | | - | - | - | - | - | - | - | |
| mf/br | *Tragelaphus oryx* | Eland | | - | 3 | 1 | - | - | 48 | 8 | |
| gr | *Syncerus caffer* | African buffalo | | - | - | 2 | - | 1 | - | - | |
| gr | *Syncerus antiquus* | Giant buffalo | | - | - | 1 | 1 | - | - | - | |
| gr | *Hippotragus leucophaeus* | Blue antelope | | - | - | - | - | - | 6 | 6 | |
| mf | *Pelea capreolus* | Grey (Vaal) rhebok | | - | - | 1 | - | 1 | 6 | 2 | |
| mf/br | *Raphicerus campestris* | Steenbok | | - | - | - | - | - | - | 1 | |
| br | *Raphicerus melanotis* | Cape grysbok | | - | - | - | - | - | 10 | 2 | |
| br | *Raphicerus* sp. | Steenbok/Grysbok | | 8 | 2 | 5 | 3 | 13 | 101 | 48 | |
| br | *Oreotragus oreotragus* | Klipspringer | | 1 | - | 2 | - | 18 | - | - | |
| mf | *Antidorcas marsupialis* | Springbok | | - | - | - | - | - | - | 1 | |
| mf | *Sylvicapra grimmia* | Grey duiker | | 1 | - | 1 | - | - | - | 2 | |
| gr | *Alcelaphini* indet. | Hartebeest/Wildebeest - | | | - | - | - | 1 | 5 | - | |
| gr | *Damaliscus/Redunca* | Bontebok/Reedbuck | - | | - | - | - | - | - | - | |
| gr | *Hippopotamus amphibius* | Hippopotamus | - | | - | - | - | - | 3 | 1 | |

**Table M in S1 File.** Dietary regime and NISP of taxa from the M1 and M2 phases at Blombos Cave (gr = grazer; mf = mixed-feeder; br = browser).

*Analysis by R.G. Klein and K. Cruz-Uribe (from Henshilwood *et al.* [38])

**Table N in S1 File.** Dietary regime and NISP of taxa from the M3 phase at Blombos Cave (gr = grazer; mf = mixed-feeder; br = browser).

| **Diet** | **Taxa** | **Common name** | **CH-CI** | **CJ** | **CK** | **CL** | **M3 (KC*)** |
| --- | --- | --- | --- | --- | --- | --- | --- |
| br | *Diceros bicornis* | Black rhinoceros | - | - | - | - | 1 |
| gr | *Equus capensis* | Cape horse | - | - | - | - | 4 |
| gr | *Redunca arundinum* | Southern reedbuck | - | 1 | - | - | 6 |
| mf/br | *Tragelaphus oryx* | Eland | 6 | 2 | 3 | 4 | 15 |
| gr | *Syncerus caffer* | African buffalo | - | - | - | - | 1 |
| gr | *Syncerus antiquus* | Giant buffalo | - | 7 | - | - | - |
| mf | *Pelea capreolus* | Grey (Vaal) rhebok | - | - | - | - | 1 |
| mf/br | *Raphicerus campestris* | Steenbok | 5 | 1 | - | 1 | 4 |
| br | *Raphicerus melanotis* | Cape grysbok | 4 | - | - | - | 3 |
| br | *Raphicerus* sp. | Steenbok/Grysbok | 64 | 34 | 8 | - | 56 |
| br | *Oreotragus oreotragus* | Klipspringer | 1 | - | - | - | - |
| mf | *Antidorcas sp.* | Springbok | 1 | - | - | - | 1 |
| gr | *Damaliscus pygargus* | Bontebok/Blesbok | 4 | - | - | - | - |
| gr | *Alcelaphus buselaphus* | Red hartebeest | 1 | 2 | - | - | - |
| gr | *Alcelaphini* indet. | Hartebeest/Wildebeest - | | - | - | - | 1 |
| gr | *Damaliscus/Redunca* | Bontebok/Reedbuck | 1 | - | - | - | - |
| gr | *Hippopotamus amphibius* | Hippopotamus | 1 | - | - | - | - |

*Analysis by R.G. Klein and K. Cruz-Uribe (from Henshilwood *et al.* [38])

| **Diet** | **Taxa** | **Common name** | **PAY** | **PAZ** | **PBA/PBB** | **PBC** | **PBD** | **PBE** | **PCA** | **Total** |
| --- | --- | --- | --- | --- | --- | --- | --- | --- | --- | --- |
| br | *Diceros bicornis* | Black rhinoceros |  |  |  | 2 |  |  |  | 2 |
| gr | *Equus* sp*.* | Zebra |  |  | 10 | 25 | 2 |  | 2 | 39 |
| gr | *Redunca fulvorufula* | Mountain reedbuck |  |  | 1 |  |  |  |  | 1 |
| gr | *Redunca arundinum* | Southern reedbuck |  |  |  | 1 |  |  |  | 1 |
| gr | *Redunca* sp. | Reedbuck |  |  | 3 |  | 1 |  |  | 4 |
| mf/br | *Tragelaphus oryx* | Eland |  |  |  |  |  | 1 | 4 | 5 |
| gr | *Syncerus antiquus* | Giant buffalo |  |  | 1 |  |  |  |  | 1 |
| mf | *Pelea capreolus* | Grey (Vaal) rhebok |  | 1 | 3 |  |  |  | 2 | 6 |
| mf/br | *Raphicerus* sp. | Steenbok/Grysbok | 1 | 1 | 1 | 8 | 7 | 5 |  | 23 |
| gr | *Ourebia ourebi* | Oribi |  |  | 1 |  |  |  |  | 1 |
| br | *Oreotragus oreotragus* | Klipspringer |  |  |  |  | 4 |  |  | 4 |
| mf | *Antidorcas cf. marsupialis* | Springbok |  |  |  |  |  |  |  | 0 |
| mf | *Sylvicapra grimmia* | Grey duiker |  |  | 2 |  | 2 |  |  | 4 |
| gr | *Damaliscus pygargus* | Bontebok/Blesbok |  | 1 | 1 | 2 |  |  | 1 | 5 |
| gr | *Damaliscus* indet. | Bles or bontebok/?D. niro |  |  | 3 |  |  |  |  | 3 |
| gr | *Alcelaphus buselaphus* | Red hartebeest |  |  | 1 | 1 | 2 |  | 1 | 5 |
| gr | *Connochaetes gnou* | Black wildebeest |  |  |  | 1 | 1 | 1 |  | 3 |
| gr | *Alcelaphini* indet. | Hartebeest/Wildebeest |  |  | 5 | 2 | 1 |  | 1 | 9 |

**Table O in S1 File.** Dietary regime and NISP of taxa at Klipdrift Shelter (gr = grazer; mf = mixed-feeder).

**Supporting Information References**

79. Jacobs Z, Duller GAT, Wintle AG. Optical dating of dune sand from Blombos Cave, South Africa: I - multiple grain data. *J Hum Evol*. 2003; 44: 599-612.
